# Presentation and response timing accuracy in Adobe Flash and HTML5/JavaScript Web experiments

**DOI:** 10.3758/s13428-014-0471-1

**Published:** 2014-06-06

**Authors:** Stian Reimers, Neil Stewart

**Affiliations:** 1 Department of Psychology, City University London, Northampton Square, London, EC1V 0HB UK; 2 Department of Psychology, University of Warwick, Coventry, UK

**Keywords:** Web, Internet, Reaction time, Response time, Display

## Abstract

Web-based research is becoming ubiquitous in the behavioral sciences, facilitated by convenient, readily available participant pools and relatively straightforward ways of running experiments: most recently, through the development of the HTML5 standard. Although in most studies participants give untimed responses, there is a growing interest in being able to record response times online. Existing data on the accuracy and cross-machine variability of online timing measures are limited, and generally they have compared behavioral data gathered on the Web with similar data gathered in the lab. For this article, we took a more direct approach, examining two ways of running experiments online—Adobe Flash and HTML5 with CSS3 and JavaScript—across 19 different computer systems. We used specialist hardware to measure stimulus display durations and to generate precise response times to visual stimuli in order to assess measurement accuracy, examining effects of duration, browser, and system-to-system variability (such as across different Windows versions), as well as effects of processing power and graphics capability. We found that (a) Flash and JavaScript’s presentation and response time measurement accuracy are similar; (b) within-system variability is generally small, even in low-powered machines under high load; (c) the variability of measured response times across systems is somewhat larger; and (d) browser type and system hardware appear to have relatively small effects on measured response times. Modeling of the effects of this technical variability suggests that for most within- and between-subjects experiments, Flash and JavaScript can both be used to accurately detect differences in response times across conditions. Concerns are, however, noted about using some correlational or longitudinal designs online.

**Electronic supplementary material:**

The online version of this article (doi:10.3758/s13428-014-0471-1) contains supplementary material, which is available to authorized users.

For nearly two decades, psychologists and behavioral scientists have used the internet, particularly the World Wide Web, to gather data for their research. Developing from crude early experiments using simple HTML forms (see Musch & Reips, 2000, for an overview of early research) to more recent complex studies replicating within- and between-subjects response time effects seen in laboratory studies (e.g., Crump, McDonnell, & Gureckis, 2013), Web-based psychological research has become mainstream. Two key factors have contributed to the rapid expansion in Web-based research. First, it has become much easier to recruit participants, using commercial panels, and allowing researchers to run experiments that might take weeks in the lab in a matter of days or hours. Second, Web-based technology has developed to allow relatively easy generation of experiments online.

The expansion in Web-based experimentation has meant that examining whether data collected online are valid and reliable is becoming an increasingly important part of the research process. In this article, we examine whether the response time data from Web-based experiments are accurate enough to be potentially useful to researchers. Before doing so, we will give a short overview of how Web-based research has developed recently.

## Participant recruitment

Early research online tended to rely on unpaid volunteers to act as participants, recruited in several ways: via emailed links to an online experiment, online advertising, referral from sites indexing Web-based psychological research, and collaborations with organizations that have large amounts of Web traffic (e.g., Kinderman, Schwannauer, Pontin, & Tai, 2013; Reimers, 2007). More recently, social networks such as twitter and Facebook have been used to recruit experimental participants. These approaches have had limited control over participant numbers, meaning that experiments have often had to remain active for long periods of time to gather enough data to test a hypothesis. Recently, many of these problems have been obviated by the emergence of crowdsourcing marketplaces such as Amazon Mechanical Turk (AMT, or MTurk elsewhere) in the US (see Mason & Suri, 2012), and reward panels such as Maximiles in the UK (see Reimers, 2009, for a brief overview). This has allowed researchers to recruit relatively reliable, demographically diverse participants, quickly, with minimum effort, and for low cost (see, e.g., Mason & Suri, 2012, for discussion of potential ethical issues around this). The use of AMT is increasing rapidly. For example, of the 63 publications in psychology and the behavioral sciences that include the phrase “mechanical turk,” listed by Web of Science, over half (33) were published between January and September 2013, the time of writing this article. Since there is increasing evidence that traditional laboratory findings are replicated in online samples (e.g., Crump et al., 2013; Kassam, Morewedge, Gilbert, & Wilson, 2011; Keller, Gunasekharan, Mayo, & Corley, 2009; Reimers & Maylor, 2005; Simcox & Fiez, 2014; Stewart, Reimers, & Harris, 2014), the use of Web-based methods and samples such as those on AMT are being increasingly used in favor of more time consuming laboratory experiments. Indeed, such is the popularity of AMT among psychology researchers, that concern has been raised about the over-participation of some workers across multiple related studies (Chandler, Mueller, & Paolacci, 2013).

## Technological development

In parallel with the development of participant recruitment techniques, the technology for conducting research online has also progressed. The earliest studies tended to use simple HTML to display text, and forms to gather data from participants (e.g., Birnbaum, 2000), with limited use of images or graphics. In contrast, survey software (e.g., Qualtrics, SurveyMonkey, Google Consumer Surveys) now allows nonprogrammers to set up studies with full randomization and random allocation of participants to conditions, as well as easily embed images and other rich content. The browser-plugin-based software Adobe Flash and Java have both been through several iterations in the past ten years, improving their capacity for handing video and other material. The arrival of the HTML5 standard now provides support for video, audio, and animation natively within the browser.

Researchers have used some of these technologies to create more user-friendly psychological testing software, such as ScriptingRT (Schubert, Murteira, Collins, & Lopes, 2013), WebExp (Keller et al., 2009), and WEXTOR (Reips & Neuhaus, 2002).

In addition to new software, the processing power of hardware has continued to follow Moore’s law, leading to an approximate doubling of computer speed every two years, meaning machines are capable of handling ever more complex dynamic stimuli and calculations.

Although it is now feasible to run a wide variety of experiments quickly and reliably online, the majority of studies, for example those listed on sites such as the Hanover College http://www.psych.hanover.edu/research/exponnet.html and http://www.socialpsychology.org/expts.htm, use untimed surveys or questionnaires, rather than variables that are captured in response times or speed–accuracy trade-offs. This may to some degree reflect the field in general, and the relative difficulty inherent in setting up response time measures online, but it is likely also to reflect a degree of skepticism about the accuracy of response time measures recorded in a Web browser online.

Although the technology and possibilities for online experimentation have developed since the earliest, text-based experiments in the mid-1990s, the discussion about the pros and cons of Web versus lab research has tended to focus on the same issues. Arguments for the use of Web-based research include the ease, speed, and cost-effectiveness of using online samples, and the more representative nature of online respondents relative to the undergraduate psychology students who typically participate in lab-based studies. Arguments against the use of Web-based research include concerns about the reliability of data, particularly with reference to repeat submissions, “junk” responding, distractions, lack of motivation, and possible variability in the way in which stimuli are presented, or responses collected, and technical variability across participants’ computer systems.

## Response time experiments online

Perhaps as a consequence of the increasing feasibility of running experiments online, there has recently been renewed interest in recording response times in online research. Although early studies were able to gain coarse measures of response times by logging the timing of server requests, most attempts at accurate response time measurement have used client-side processing, that is, running code and measuring times on the user’s own machine, traditionally using browser plugins such as Java and Adobe Flash, but more recently also JavaScript, which is natively supported by browsers. We give a brief overview for each now.

### Java

Java is a powerful, flexible development platform, which runs on a wide range of computing devices. In Web browsers, Java runs as an ostensibly sandboxed client-side virtual machine plugin. For users that have the Java plugin installed, opening a webpage that contains a Java applet will initialize Java, which will then run the applet. The sandboxed nature of the Java virtual machine means that any code run should be limited in what it is permitted to do with a user’s machine, such as restricting access to files and prohibiting installation of software. Advantages of Java include the fact that is it a well-established programming language, and it is theoretically platform-independent. It is also used extensively on non-PC devices: For example, smartphone Android apps are written in a customized version of Java, with about 675,000 apps and 25 billion downloads (Rosenberg, 2012). Disadvantages include the fact that many browsers do not have the plugin, the relatively long initialization time, the number of different versions of the Java runtime, and the complexity of the language, which makes designing simple user interfaces less than straightforward. A further downside is that in 2013, serious security issues in Java were uncovered, prompting the US Department of Homeland Security to recommend that users uninstall Java to maintain security. Browsers now often present a security warning before Java applets are displayed, meaning that applets no longer start automatically.

### Flash

Adobe Flash is an authoring platform primarily used for the development of Web-based games, animations and interactive tools. Flash is installed on a large majority of Web-facing computers. Like Java, Flash Player runs as a client-side sandboxed virtual machine. Advantages of Flash include the ubiquity of its plugin; the complexity of the programs that can be implemented, particularly since the advent of ActionScript 3; its ostensible platform independence; and the fact that the software has been designed specifically for authoring interactive materials on the Web. Disadvantages include the proprietary nature of the software, its absence from Apple touchscreen devices such as iPhones and iPads, and its own potential security issues.

### HTML5, CSS3, and JavaScript

The combination of HTML5, JavaScript, and CSS3 (hereafter abbreviated to HTML5) now provides an alternative to Java and Flash. HTML, JavaScript, and CSS3 are three different technologies that work together to render webpages or apps. The HTML component provides the static content like text, pictures, sounds and video. CSS3 is used to style the content, setting fonts, colors, and positioning. JavaScript is used to make the content dynamic, hiding and showing elements of the HTML, making them draggable or clickable, and so on. The HTML5 alternative is provided in all major browsers on all desktop and mobile platforms, used extensively on the Web (e.g., behind Wikipedia and Gmail), and provides video, audio, and animation support not previously available without a plugin. Psychologists are also beginning to make use of HTML5 as a way of running traditional lab-based studies across a range of devices and Web browsers (see, e.g., www.ertslab.com). Applications both for Windows and iOS operating systems can be developed in HTML5. Since HTML5 is natively provided in most major browsers without plugins, there are no security warnings to the user. Libraries like jQuery have reduced cross-browser differences that were problematic for earlier HTML and JavaScript implementations. HTML5 provides an open and free (cf. proprietary Flash) development environment. On the downside, unlike Java and Flash, it is easy to view the source code, which means that skilled participants could reverse engineer an experiment to work out, for example, the experimental manipulations, or to more easily program an automated “bot” to respond multiple times in the experiment. Relative to Flash, HTML5’s capabilities are slightly more limited (e.g., the HTML5 standard does not support full-screen presentation or streaming video, although many current HTML5-compatible browsers do), and browsers vary in the elements of HTML5 that they support, with many older, but still used, browsers—such as Internet Explorer 8—supporting relatively few HTML5 elements. Even where elements are supported, differences in appearance and functionality can be seen across browsers. However, with Adobe’s 2011 announcement that they are no longer developing Flash for mobile devices and instead will contribute to HTML5 (Winokur, 2011), as well as the decline in Flash objects on top websites, an increasing switch from Flash to HTML5 appears likely.

All three software approaches mentioned above have been used in an attempt to examine the feasibility of collecting response time data from online behavioral experiments. In general, the results have been fairly similar across implementation, although it is rare to see examples of direct comparisons across different implementations. Research has generally taken two approaches. The first approach has involved attempts to replicate well-established response time effects online; to see whether the same patterns of results can be found. The second has been to use specialist hardware to examine how accurately browser-based software can display stimuli and record response times.

### Comparisons of Web- versus lab-based experimental results

In the past few years, several authors have attempted to compare response time data between online and lab-based collection, or replicate classic response-time-based findings online. We (Reimers & Stewart, 2007) compared participant response times within subjects, using a C++-based millisecond-accurate response system, and the same experiment coded in Flash and run on a browser. Participants completed the same simple and choice reaction time experiments using the accurate system, using Flash in a browser run on a laboratory computer, and using Flash on their own computer in their own time. We found that response times on the Flash system were recorded as around 40 ms longer than those on the accurate system, but that relatively little random noise was introduced in the Flash conditions. We also examined response times using cellphones running Adobe Flash Lite, which gave much less reliable data in some cases (Reimers & Stewart, 2008). More recently, Schubert et al. (2013) compared Stroop task performance between laboratory and Web-based Flash, and found broadly similar performance.

Rather than directly compare experimental results across Web and lab implementations, a number of recent studies have attempted to replicate well-established response-time-based phenomena online. The rationale behind this is that if well-established, “classic” results can be replicated, then the details of differences in timings online are relatively unimportant. Most published replication attempts have shown very similar results to those from lab-based studies. In one of the earliest examples, Reimers and Maylor (2005) used Adobe Flash to examine the effects of age on task-switching performance, finding largely similar results to those from laboratory studies, where data existed. Similarly, Keller at al. (2009) replicated a response time effect in a psycholinguistic experiment; Simcox and Fiez (2014) replicated flanker and lexical decision effects online. In perhaps the most extensive set of replication attempts, Crump et al. (2013) used AMT to attempt to replicate Stroop, switching, flanker, Simon, Posner cueing, attentional blink, subliminal priming, and category learning tasks. The authors replicated the majority of the effects, including a small 20-ms visual cueing effect. The two situations in which effects could not be replicated were for short-duration masked priming (with prime durations of 64 ms or less), and for category learning (which was not response-time-based, and appeared to be down to motivational issues in the participants).

### Direct measurement of Web-based timing accuracy

The second strand of relevant research has examined the timing performance of Web-based testing setups using specialist software or hardware. One of the earlier attempts to examine system performance for browser-based experiments was by Keller et al. (2009). They looked specifically at the Java-based experimental software WebExp for measuring apparent response times. They used keypress repetitions as a known response time, noting that when a key on a keyboard is pressed and held down, after a while the operating system generates repeated keypresses with a known and adjustable interval between them. Since all of the intervals are known, keypress repeats can be used to assess the accuracy of a piece of experimental software’s timings. For example, if an experimenter sets the keystroke repetition rate to 300 ms, he or she can generate a stream of keypresses with a known 300-ms interval between them, and examine what the software measured as the interval, giving a measure of timing accuracy.

A similar software-based approach was used by Simcox and Fiez (2014), who used a script running on the test machine to generate a stream of keypresses with fixed intervals between them and measured the accuracy with which these intervals replicated across repeated runs of the same script. Like Keller et al. (2009), they found very good timing performance.

Although the findings of Keller et al. (2009) and Simcox and Fiez (2014) are informative, there are two reasons why they may lead to underestimation of timing errors. The first is that in both cases the input keypresses are generated by the same system as is used for recording the keypress timing accuracy. Although this is an understandable approach to take, it means that both stimulus generation and response time recording rely on the same system clock. If the clock information available to generation and recording software is consistent but inaccurate, there is no way to detect this. The second potential problem is that software-based approaches tend to measure the gaps between multiple keypress events, rather than the gap between presentation of a stimulus and a keypress, which is typically the focus of behavioral research. In a system in which, for example, the testing software recorded all events as occurring 100 ms after they actually did, the gaps between keypresses would remain the same and would be measured accurately, but response times to stimuli would be measured as 100 ms longer than they actually were. Just looking at the gap between keypresses would risk giving a false impression that the system was recording responses accurately. For that reason, a better setup would be to use external hardware to respond to stimuli displayed on the screen with a fixed, known response time, and compare with the response times measured by the software under consideration. In other words, using hardware to mimic a human participant, but with known response parameters.

We are aware of two studies that have done this. The first was conducted by Neath, Earle, Hallett, and Surprenant (2011) on two Apple Macintosh computers. They used a photodiode to detect visual stimuli presented on the screen, and then triggered a solenoid to press a key after a known period of time. The authors examined Web-based software such as Java, JavaScript, and Flash, among other things. They found increased variability and overestimation of response times, and some quantizing of response times with Flash.

The second study was conducted by Schubert et al. (2013), using their bespoke Flash-based testing setup ScriptingRT. They used a photodiode attached to the screen to generate responses either directly, or using a solenoid to press a key on a standard keyboard. Their main focus was on comparing response time variability using ScriptingRT with that using testing software such as E-Prime and DMDX. They found somewhat higher variance in their Flash-based setup, although it was still small, relative to other testing software, and 10- to 35-ms longer measured response times. Since the response system was not validated, it was not possible to estimate the absolute overestimation of response times using ScriptingRT, but the overestimation was not markedly longer than using traditional testing packages.

Finally, in contrast to research that has examined the accuracy of response times in online experiments, a much smaller literature has examined the accuracy of presentation durations online. Although for most research, the precise control over stimulus durations is not vital, several research areas—such as, for example, masked priming, iconic memory, and change blindness—require more accurate presentation timings. Existing research suggests that stimulus duration accuracies are reasonably good. For example, Simcox and Fiez (2014) used a photodiode to compare actual and intended visual presentation durations under Adobe Flash, and found reasonably accurate presentations. Schubert et al. (2013) found that their Flash-based stimulus presentation was around 24 ms longer than intended, across three presentation durations.

### Research rationale

This evidence using specialist hardware suggests that it might be feasible to run timing-sensitive experiments online, both those that require accurate stimulus presentation durations, and those that measure response times online. However, even the most extensive existing studies (Neath et al., 2011; Schubert et al., 2013) have been run on just one or two systems, meaning the although we have some idea of how much within-system variability may arise from running an experiment in a Web browser, we have no idea how much between-system variability exists.

For within-subjects experiments, or tacitly within-subjects experiment in which participant performance is compared to a baseline condition (e.g., switch costs, Simon effect magnitude, priming magnitude, Stroop effect), between-system variability is not an issue. Measured Stroop effects or switch costs would be the same even if measured response times were much longer than actual response times. However, within-system variability would be an issue. If a system introduced large amounts of noise into each measured response time, the power to detect differences between conditions would be reduced. This could be compensated for by using a larger number of trials (see, e.g., Damian, 2010, for a discussion).

For between-subjects experiments, between-system variability is more of an issue, as it introduces random noise into estimates of group means for response times in different conditions. Large variability between systems would make it harder to detect differences between groups. Without having an estimate of between-system variability, it is not possible to estimate how much power would be lost in a given Web-based response time experiment as a result of variability in performance across different machines.

Of more concern, there appear to be two areas in which between-system variability risks leading to false positive findings: Correlational studies and longitudinal studies. If, for example, older operating systems measured much longer response times than newer ones, and older people tended to use older operating systems, spurious age effects might arise. Similarly, if, say, the browser Firefox is used by a greater proportion of men than other browsers, and it happens to record faster response times, then spurious sex differences could be obtained. For longitudinal data, if upgrades to operating systems or hardware mean that overestimation of response times decreases, it may appear that a cohort is getting faster (or not getting slower) with age.

Finally, we note that we are only referring to simple experimental designs that present individual stimuli, and require no specialist hardware. For experiments that require synchronization of input or output across modalities or different pieces of hardware, small timing differences could have substantial effects on the results.

The aim of the research reported here was therefore to attempt to quantify the extent to which display durations and measured response times may vary across participants for nonbehavioral reasons. To do this, we use specialist hardware to measure stimulus display durations accurately, and generate responses to stimuli with fixed, known latencies.

In Study 1, we used four different systems to examine systematically the effects of implementation (Flash vs. HTML5), browser type (Internet Explorer, Firefox, or Chrome), and duration or stimulus or response latency (short, medium, or long). For the least powerful system in this group, we also examine the effect of concurrent processor load on timing performance. In Study 2, we use a single duration and latency, on a single browser, to examine variability across a further 15 systems running the same code.

We constrained this analysis just to Windows PCs. We found that 85 %–90 % of participants who complete our online experiments do so using a Windows PC. Most of the remainder use a Mac. Very few (<1 %) participants use other operating systems, such as Linux. We refer readers interested in Mac response time measurements to Neath et al.’s (2011) article.

The unique contributions of this article include (a) a direct comparison of the timing properties of two popular ways of running online experiments—Flash and HTML5; (b) a systematic test of stimulus presentation and response recording accuracy across different browsers and durations; and (c) an examination of system-to-system variability and exploration of the potential causes of that variability.

## Study 1

The aim of Study 1 was to examine the ways and extent to which timings in online research might be subject to noise. We were interested both in the variability in timing within a given computer system, and across systems. We used a Black Box Toolkit Version 2 (www.blackboxtoolkit.com; see also Plant, Hammond, & Turner, 2004, for an introduction to its Version 1 predecessor) to examine timing accuracy. The toolkit is a piece of hardware designed for use by psychologists and neuroscientists to record stimulus presentation timings and respond with known response times. Specifically it can detect and record visual and auditory stimulus onset and offset with sub-millisecond accuracy, using opto-detectors attached to a computer monitor, and microphones placed in front of a computer’s loudspeakers. It can also generate events with precise latencies, such as sounds and switch closures. By dismantling a keyboard or mouse, and attaching wires on either side of a key or mouse button, the switch closure can then generate an event like a keypress or mouse click. This allows the toolkit to generate responses to stimuli with a known, precise latency. For example, it can generate a keypress exactly 300 ms after a stimulus appears on a screen (although this setup does not take into account key travel time or switch registration time that would be a part of a real human’s response time). The testing software can measure the apparent response time, allowing examination of how it deviates from the actual response time.

For these tests, we use a simple setup, coded as simply as possible in Flash (ActionScript 3) and HTML5, presenting a white square on a black background (to maximize contrast and aid detection of stimulus onset). For the visual display duration test, the square remains on the screen for a period of time specified in the Flash or JavaScript code. For the response time test, the square remains onscreen until a keypress response is recorded. The materials, code, and data from these studies are available as Supplementary Materials.

### Visual display duration (VDD)

This procedure measures the actual duration of visual stimuli, to allow comparison with intended duration. Once started, Flash or JavaScript code presents a white square on a black background for a fixed period of time, after which it disappears. This is followed by a 500-ms intertrial interval (ITI) and the next presentation. A total of 100 trials were presented for each condition. Three different display durations were set in Flash and JavaScript: 50, 150, and 500 ms. We used 50 ms to examine the potential for running, for example, masked priming experiments online; 150 ms for stimuli that might be presented for a single fixation; and 500 ms for more conventional stimuli that allow multiple fixations.

The Flash version of this procedure was coded in ActionScript 3 with a presentation rate of 60 frames per second (fps). It used the Timer() procedure linked to an event listener to specify how long after the square was presented the square should be hidden again. [We separately used the alternative setInterval() method in initial testing, which gave similar results.]

The HTML5 implementation was coded with the jQuery library. The square was an HTML5 <div> element formatted in CSS3. The square was hidden and shown using JavaScript using the hide() and show() methods. We provided callback functions to these methods to chain hiding and showing. For example, when the show() method was called it displayed the square and ran a callback function that used the hide() method to hide the square after the display duration had elapsed. And when the hide() method was called it hid the square and ran a callback function that used the show() method to show the square after the ITI has elapsed. Timing was provided by the delay() method.

Actual durations were recorded using the black box toolkit, using its inbuilt Digital Stimulus Capture procedure. An opto-detector was attached to the screen to detect stimulus presentation durations. The toolkit recorded only onsets and offsets of stimuli, so a brightness threshold had to be set to mark onset and offset. Thresholds set for triggering varied according to ambient light and monitor brightness and contrast, but were between 100 (78 %) and 115 (90 %) on the toolkit’s internal scale.

### Response time measurement

This procedure simulates a participant with a known response time. Once started, Flash/HTML5 code presents a white square on a black background (as in VDD), starts a timer, and awaits a keypress response. On receiving a response, the timer is stopped, the time elapsed is recorded, the white square disappears, and a 500-ms ITI begins, followed by the next trial, for a total of 100 trials. After 100 trials, the recorded response times for all trials are displayed by the Flash/HTML5 code on the screen and can be copied and pasted for analysis.

Here, we had the black box toolkit respond with response times of precisely 150, 300, and 600 ms. We used 150 ms as the lower bound for response times in a simple reaction time experiment; 300 ms as the lower bound for response times in a basic choice reaction time experiment; and 600 ms for a more typical response time in a speeded categorization or judgment experiment. Since response variability is largely in proportion to response time, the types of experiment that are most susceptible to the effects of hardware or software variability are those with the shorter response times.

As before, the Flash version of this procedure was coded in ActionScript 3 with a frame rate of 60 fps. It used the getTimer() procedure to record the times at which the stimulus was presented and the keypress detected. The HTML5 implementation detected keypresses by binding an event-handler function to the JavaScript “keydown” event. Timing of the onset of the square was recorded using the jQuery function $.now(), which provides the time in milliseconds in a callback function for the javascript show() method. Timing of the key press was recorded with $.now() in the “keydown” event handler.

The tests were run using the toolkit’s inbuilt Digital Stimulus Capture and Response procedure. As before, an opto-detector was attached to the screen to detect stimulus onset. To allow the toolkit to generate a keypress, a standard Dell USB keyboard (model SK-8115) was dismantled, and wires placed on top and bottom contacts for the space bar. These wires were connected to the active switch closure terminals in the toolkit’s breakout board. The toolkit was programmed to generate a switch closure a precise, constant period of time after stimulus onset. This would be detected by the system running the Flash or HTML5 experiments as a space bar press.

### Hardware and operating systems

We ran the tests on the following PC systems (fuller system information can be found in Table 6 below):

**Table 1:** Top: Differences between intended and actual presentation (positive = longer duration than intended) on a Dell OptiPlex 760 desktop computer for intended durations of 50, 150, and 500 ms. Bottom: Differences between actual and measured response times (positive = overestimation) on a Dell OptiPlex 760 desktop for actual response times of 150, 300, and 600 ms

	Flash			HTML5		
	MSIE	Firefox	Chrome	MSIE	Firefox	Chrome
*Presentation Time*						
*50 ms*						
Mean (*SD*)	+15.2 (7.9)	+14.6 (8.5)	+16.3 (7.9)	+36.5 (1.7)	+22.3 (9.6)	+4.3 (9.5)
Range	+3, +36	+4, +25	–13, +26	+20, +37	+1, +35	–12, +25
*150 ms*						
Mean (*SD*)	+6.1 (8.3)	+9.4 (8.2)	+3.0 (8.4)	+25.2 (8.3)	+4.9 (14.5)	–1.9 (10.2)
Range	–1, +16	0, +19	–5, +29	+17, +33	–15, +35	–16, +19
*500 ms*						
Mean (*SD*)	+0.6 (1.7)	–0.5 (1.7)	+1.1 (7.0)	+17.1 (1.9)	–0.2 (5.4)	+7.0 (8.4)
Range	0, +17	–1, +16	–16, +17	+16, +34	–18, +32	–15, +18
*Response Time*						
*150 ms*						
Mean (*SD*)	+26.7 (7.8)	+32.9 (7.3)	+28.8 (8.0)	+33.6 (7.5)	+45.0 (7.6)	+38.7 (12.4)
Range	+6, +38	+22, +83	+21, +52	+21, +53	+22, +56	+7, +67
*300 ms*						
Mean (*SD*)	+27.3 (8.1)	+35.3 (8.2)	+29.6 (7.4)	+33.2 (8.4)	+42.8 (6.7)	+43.4 (12.9)
Range	+12, +44	+19, +50	+12, +44	+12, +44	+20, +64	+8, +69
*600 ms*						
Mean (*SD*)	+28.1 (8.0)	+36.7 (5.1)	+28.5 (7.9)	+34.7 (8.7)	+43.9 (9.8)	+41.8 (16.4)
Range	+9, +41	+27, +48	+9, +41	+24, +56	+6, +70	+10, +71

MSIE, Microsoft Internet Explorer

**Table 2:** Deviations from intended stimulus durations (top) and actual response times (bottom) using a Sony Vaio under low load

	Flash			HTML5		
	MSIE	Firefox	Chrome	MSIE	Firefox	Chrome
*Stimulus Duration*						
*50 ms*						
Mean (*SD*)	+17.2 (9.4)	+16.5 (9.0)	+19.8 (9.7)	+46.4 (6.6)	+18.7 (8.3)	+16.8 (7.6)
Range	–7, +45	–6, +44	–5, +47	+27, +63	+8, +31	+8, +30
*150 ms*						
Mean (*SD*)	+27.3 (5.9)	+27.5 (3.0)	+20.5 (8.7)	+39.7 (10.1)	+19.5 (8.4)	+15.7 (7.4)
Range	–14, +32	+12, +3	+9, +39	+26, +98	+8, +31	+8, +31
*500 ms*						
Mean (*SD*)	+11.0 (4.2)	+11.2 (3.2)	+11.5 (2.7)	+32.3 (7.1)	+20.0 (8.6)	+14.9 (6.3)
Range	–7, +15	–6, +15	–4, +15	+12, +48	+10, +43	+10, +31
*Response Time*						
*150 ms*						
Mean (*SD*)	+32.4 (8.6)	+41.9 (6.1)	+47.0 (6.7)	+41.7 (9.0)	+57.6 (7.0)	+45.5 (5.9)
Range	+21, +53	+29, +56	+31, +62	+21, +69	+40, +71	+32, +60
*300 ms*						
Mean (*SD*)	+41.9 (7.4)	+41.8 (6.6)	+46.7 (7.0)	+45.3 (8.5)	+60.0 (32.3)	+46.1 (6.9)
Range	+26, +57	+28, +58	+31, +64	+28, +60	+40, +373	+28, +61
*600 ms*						
Mean (*SD*)	+43.9 (6.9)	+43.4 (6.5)	+48.1 (6.6)	+51.2 (7.4)	+56.4 (4.8)	+50.0 (36.3)
Range	+26, +57	+30, +57	+34, +62	+40, +57	+43, +63	+31, +405

MSIE, Microsoft Internet Explorer

**Table 3:** Deviations from intended stimulus durations (top) and actual response times (bottom) using a Sony Vaio under high load

	Flash			HTML5		
	MSIE	Firefox	Chrome	MSIE	Firefox	Chrome
*Stimulus Duration*						
*50 ms*						
Mean (*SD*)	+15.6 (8.9)	+26.6 (41.0)	+19.9 (15.1)	+50.6 (9.3)	+24.2 (12.6)	+22.1 (8.3)
Range	–7, +31	–7, +346	–7, +96	+27, +64	–6, +63	+9, +46
*150 ms*						
Mean (*SD*)	+23.8 (8.3)	+23.7 (23.9)	+23.5 (17.1)	+50.7 (11.7)	+24.5 (10.2)	+19.6 (9.3)
Range	–8, +45	–7, +143	–7, +96	+26, +93	+8, +62	+8, +45
*500 ms*						
Mean (*SD*)	+10.8 (5.6)	+22.8 (20.7)	+15.1 (13.4)	+43.6 (11.3)	+25.6 (14.5)	+19.7 (9.2)
Range	–7, +29	–4, +128	–23, +62	+26, +77	+9, +94	+8, +47
*Response Time*						
*150 ms*						
Mean (*SD*)	+37.3 (6.9)	+45.2 (29.4)	+46.7* (11.2)	+42.9 (10.5)	+55.6 (10.7)	+44.0 (5.8)
Range	+23, +58	+22, +283	+28, +113	+21, +69	+35, +100	+29, +62
*300 ms*						
Mean (*SD*)	+37.1 (7.4)	+48.2 (31.0)	+47.6 (12.2)	+44.9 (10.5)	+52.0 (7.2)	+42.2 (6.4)
Range	+24, +51	+22, +234	+30, +89	+28, +75	+36, +71	+24, +57
*600 ms*						
Mean (*SD*)	+39.0 (7.3)	+48.9 (34.7)	+48.9 (11.6)	+44.8 (15.5)	+53.9 (7.4)	+43.8 (6.7)
Range	+24, +68	+24, +280	+31, +108	+25, +165	+40, +102	+27, +61

MSIE, Microsoft Internet Explorer. *This was run initially and showed a slightly worse performance. Unfortunately the black box toolkit logs were inadvertently discarded, so it was rerun

**Table 4:** Deviations from intended stimulus durations (top) and actual response times (bottom) using a Dell OptiPlex 790

	Flash			HTML5		
	MSIE	Firefox	Chrome	MSIE	Firefox	Chrome
*Stimulus Duration*						
*50 ms*						
Mean (*SD*)	+26.1 (9.4)	+10.1 (9.7)	+21.5 (9.0)	+24.7 (6.1)	+20.5 (8.3)	+19.1 (9.7)
Range	+10, +61	–8, 29	–5, +29	+8, +29	+8, +29	–8, +42
*150 ms*						
Mean (*SD*)	+18.8 (9.2)	+10.5 (3.5)	+16.6 (10.7)	+24.4 (13.3)	+18.4 (8.4)	+11.8 (4.3)
Range	+11, +46	–8, +13	–6, +44	–5, +46	+10, +29	–6, +29
*500 ms*						
Mean (*SD*)	+21.4 (9.7)	+10.6 (4.6)	+10.7 (4.6)	+10.1 (7.6)	+22.3 (8.1)	+12.6 (4.1)
Range	–7, +46	–6, +13	–6, +13	–7, +30	+11, +30	–4, +30
*Response Time*						
*150 ms*						
Mean (*SD*)	+77.0 (7.8)	+79.6 (13.5)	+88.2 (8.9)	+77.9 (6.6)	+89.6 (9.5)	+82.8 (10.1)
Range	+68, +84	+60, +190	+70, +103	+65, +97	+72, +113	+65, +101
*300 ms*						
Mean (*SD*)	+76.7 (9.3)	+81.4 (8.5)	+85.6 (6.7)	+84.0 (7.8)	+88.8* (6.4)	+88.0 (8.1)
Range	+58, +90	+70, +100	+71, +101	+67, +104	+77, +105	+63, +96
*600 ms*						
Mean (*SD*)	+75.6 (8.0)	+81.2 (5.7)	+81.5 (7.8)	+82.5 (6.8)	+90.2 (6.1)	+75.5 (7.6)
Range	+55, +87	+70, +100	+67, +99	+70, +100	+71, +111	+63, +84

MSIE, Microsoft Internet Explorer. *The first trial of this run recorded a response time of 81 ms, which was excluded. All other recorded response times were >350 ms

**Table 5:** Deviations from intended stimulus durations (top) and actual response times (bottom) using a Dell OptiPlex 9010 machine

	Flash			HTML5		
	MSIE	Firefox	Chrome	MSIE	Firefox	Chrome
*Stimulus Duration*						
*50 ms*						
Mean (*SD*)	+33.1 (7.8)	+19.5 (6.2)	+17.6 (4.7)	+27.9 (10.3)	+18.5 (4.0)	+19.6 (5.0)
Range	+18, +68	+18, +51	+1, +35	+1, +51	+1, +35	+18, +35
*150 ms*						
Mean (*SD*)	+21.9 (8.5)	+16.6 (4.6)	+15.0 (0.2)	+29.3 (13.1)	+17.3 (6.2)	+16.1 (8.7)
Range	+14, +49	+15, +32	+14, +15	+15, +49	–1, +32	–2, +48
*500 ms*						
Mean (*SD*)	+27.3 (8.7)	+15.6 (7.0)	+14.6 (0.6)	+13.9 (9.5)	+19.7 (7.9)	+15.9 (4.3)
Range	+14, +49	–2, +48	+14, +15	–2, +32	+14, +31	+15, +32
*Response Time*						
*150 ms*						
Mean (*SD*)	+65.8 (9.9)	+76.2 (6.0)	+77.5 (8.2)	+77.8 (6.8)	+90.5 (7.8)	+76.4 (8.1)
Range	+52, +84	+60, +90	+64, +98	+63, +94	+75, +102	+65, +87
*300 ms*						
Mean (*SD*)	+69.3 (8.3)	+75.0 (5.2)	+79.0 (6.1)	+80.8 (7.1)	+95.9 (7.3)	+78.8 (4.8)
Range	+58, +90	+70, +90	+69, +100	+65, +95	+81, +110	+61, +95
*600 ms*						
Mean (*SD*)	+71.3 (8.9)	+77.3 (5.5)	+87.9 (7.2)	+126.3 (8.9)	+92.9 (10.7)	+88.4 (8.0)
Range	+55, +87	+60, +80	+70, +102	+110,+149	+72, +109	+78, +99

MSIE, Microsoft Internet Explorer

**Table 6:** Comparison of response times (RTs) and display performance for all machines included in the test, for 300-ms RTs and 150-ms presentation durations

System	OS	CPU (speed)	RAM	Graphics (speed)	Monitor	Deviation From Actual RT (ms)		Deviation From Specified Display Duration (ms)	
						Flash	HTML5	Flash	HTML5
**Full Test**									
Dell OptiPlex 760	XP	E2220 (1351)	3Gb	HD2400 Pro (134)	Dell P2211H	+30.7 (7.9)	+39.8 (9.3)	+6.2 (8.3)	+9.4 (11.0)
Dell OptiPlex 9010	Win 7	i3-3220 (4237)	8Gb	Intel HD 2500 (~225)	Dell P2211H	+74.4 (6.5)	+85.2 (6.4)	+17.8 (4.5)	+20.9 (9.3)
Sony Low Load	XP	Atom N280 (300)	1Gb	Intel GMA 250 (~25)	Own	+43.4 (7.0)	+45.7 (7.7)	+25.1 (5.9)	+25.0 (8.6)
Sony High Load						+44.3 (16.9)	+46.4 (8.0)	+23.7 (16.4)	+31.6 (10.4)
Dell OptiPlex 790	Win 7	i3-2100 (3596)	4Gb	Intel HD 2000 (~200)	Sharp LLT15A4B	+81.2 (8.2)	+86.9 (7.4)	+15.3 (7.8)	+18.2 (8.7)
**Concise Test**									
Toshiba L300-1BV	Vista	T1600 (999)	2Gb	GMA 4500M (~50)	Own	+67.5 (7.4)	+68.2 (15.4)	+30.7 (9.6)	+20.0 (4.2)
Toshiba L955-10N	Win 8	i3-3227U (2587)	4Gb	Intel HD 4000 (465)	Own	+55.1 (8.5)	+74.8 (7.1)	+24.8 (9.3)	+32.5 (13.1)
Toshiba L500-1XJ	Win 7	T3100 (1153)	3Gb	GMA 4500M (~50)	Own	+74.0 (8.3)	+95.4 (7.5)	+21.9 (8.4)	+23.7 (7.0)
RM Expert 3040Ma	Win 7	E7500 (1871)	4Gb	Intel GMA 4500 (~50)	Hanns.g HW191D	+62.5 (6.4)	+67.0 (7.3)	+34.9 (7.1)	+35.8 (10.7)
RM Expert 3040Mb	Win 7	E7500 (1871)	4Gb	Intel GMA 4500 (~50)	Hanns.g HW191D	+67.9 (8.2)	+76.4 (8.0)	+26.4 (8.9)	+36.5 (12.7)
Asus K40C/K50C	Win 7	Cel. D 220 (402)	2Gb	SIS Mirage 3 (~15)	Own	+70.8 (12.4)	+83.6 (7.3)	+24.3 (8.7)	+36.2 (9.4)
HP Pavilion g6	Win 7	i5 M 480 (2451)	4Gb	Intel HD (Core i5) (~75)	Own	+92.6 (7.1)	+89.2 (8.5)	+13.3 (10.3)	+11.8 (5.4)
RM Desktop 320a	Win 7	i3-3220 (4237)	4Gb	Intel HD 2500 (~250)	Hanns.g HW191D	+75.1 (8.4)	+79.3 (7.6)	+23.4 (8.5)	+18.5 (5.8)
Toshiba L300-1G8	Vista	T3400 (1108)	4Gb	GMA 4500M (~50)	Own	+64.6 (6.6)	+78.9 (8.1)	+11.3 (6.2)	+20.4 (8.5)
RM Desktop 320b	Win 7	i3-2100 (3604)	4Gb	Intel HD 2000 (~200)	Hanns.g HP191DJ0	+32.0 (8.5)	+61.3 (11.0)	+24.8 (9.2)	+22.5 (14.1)
RM Desktop 310	Win 7	i7-3770 (9426)	4Gb	GeForce GT630 (722)	Hanns.g HP198DJB	+67.9 (8.3)	+75.4 (8.0)	+24.9 (8.1)	+25.5 (8.3)
RM MINIPC 214	Win 7	i7-3770 (9426)	16Gb	Intel HD 4000 (465)	Philips 226V	+71.4 (7.6)	+81.6 (7.7)	+18.5 (4.8)	+27.6 (8.3)
Dell OptiPlex 780	Win 7	E7500 (1871)	4Gb	Intel GMA 4500 (~50)	Dell P2210	+74.0 (6.8)	+73.9 (7.7)	+18.3 (4.2)	+32.4 (8.5)
Dell OptiPlex	Win 7	i7-2600 (8312)	8Gb	Intel HD 2000 (~200)	Dell P2210	+67.4 (9.1)	+62.5 (8.6)	+21.2 (10.8)	+20.3 (7.9)
Toshiba R700-15U	Win 7	i3 M 350 (1911)	2Gb	Intel HD Core i3 (~75)	Own	+75.2 (8.2)	+99.7 (7.1)	+23.4 (9.8)	+30.5 (12.9)

Averaged across browsers for those tested on all three browsers, and across three runs of 100 trials for those tested on a single browser. CPU speeds and graphics speeds where available, were obtained from www.cpubenchmark.com. Other graphics speeds were estimated from comparisons with graphics cards with known speeds, from www.notebookcheck.net

- ***Dell OptiPlex 760 Desktop PC*** This was a machine running Windows XP that had until recently been used in an academic’s office, but had been replaced as its processing capacity was limited.
- ***Sony Vaio VPCW11S1E netbook***, ***low load*** This was a four-year-old netbook running Windows XP as an example of a very low-end computer that might be used to run an experiment.
- ***Sony Vaio VPCW11S1E netbook***, ***high load*** This setup examined what we defined as a boundary case for Web-based response time testing: An obsolescent machine with slow processor and low memory, running multiple applications during the test. For this test, we used the same netbook as above; this time before running the tests, we started a Skype video call (transmitting but not receiving video), and played a YouTube video in another—nonvisible—browser tab. According to the task manager application, this took CPU use up from less than 10 % in the low load configuration to 60 %–100 %. The Skype call and video played throughout the display and response time measurement process.
- ***Dell OptiPlex 790 Desktop PC*** This PC is the standard build of machine used in City University’s undergraduate research labs.
- ***Dell OptiPlex 9010 Desktop PC*** This PC is used for analysis and modeling work, so has a higher specification than the standard testing machines.

### Test results

#### Dell OptiPlex 760

These results are given in Table 1. It appears that, in general, within-machine variability was very low, both for display duration and response time measures. Display durations were longer than those intended in some cases, particularly for the shorter (50- and 150-ms) durations. Overall, there were minimal effects of implementation or browser type, with the exception perhaps of longer display durations for HTML5 and Internet Explorer.

#### Sony Vaio Netbook, low load

These results are given in Table 2. As before, variability was generally low, with a couple of exceptions in the HTML5 response time conditions, both of which stem from a single outlying long measured response time. Again, as before, measured display durations and response times were largely unaffected by browser and implementation, except again for longer durations in the HTML5/Internet Explorer combination.

#### Sony Vaio Netbook, high load

These results are given in Table 3. The effect of load on timing in this machine was relatively small. Response times were slightly longer, and variability rather larger (often caused by a small number of very long durations) than in the low-load condition.

#### Dell OptiPlex 790

These results are given in Table 4. Although the means and *SD*s for stimulus presentation are relatively good, response times are overestimated by around 80 ms. Despite these large overestimations, standard deviations are small.

#### Dell OptiPlex 9010

These results are given in Table 5. The results are fairly similar to those from the other Windows 7 machine, in Table 4. Again, although standard deviations are small, overestimation of response times was around 80 ms. The results could also be compared with those from the Dell OptiPlex 760 running Windows XP, in Table 1, since both used the same monitor, removing a source of variance. The standard deviations are similar, but response times are around 30 ms longer on this Windows 7 machine.

### Display duration deviations

Overall, the actual time for which a stimulus was displayed was significantly longer than the specified duration. The size of this overlength was around 20 ms. For the majority of the tests, there appears to be an interaction between browser and implementation. Overlong display durations under HTML5 were larger in Internet Explorer than Firefox or Chrome, by around 20 ms, although this effect was smaller for longer durations and for more powerful machines. For a given duration, the maximum difference in means between browsers running Flash was around 5 ms.

### Response time overestimations

In all cases, response times were overestimated. We found no obvious systematic effect of browser type. It did appear that response times were overestimated more in the HTML5 implementation, although this effect was relatively small. However, the clearest effect was in variability across systems. It appeared that the older systems running Windows XP, despite being much less powerful than their newer, Windows 7 counterparts, were much more accurate in their response time measurement. The two Windows 7 machines measured response times that were 30–40 ms longer than the XP machines. It is, of course, possible that the difference would have been larger had we been able to control for processor speed and graphics capability.

### Distribution of display and response times

**Fig. 1:**
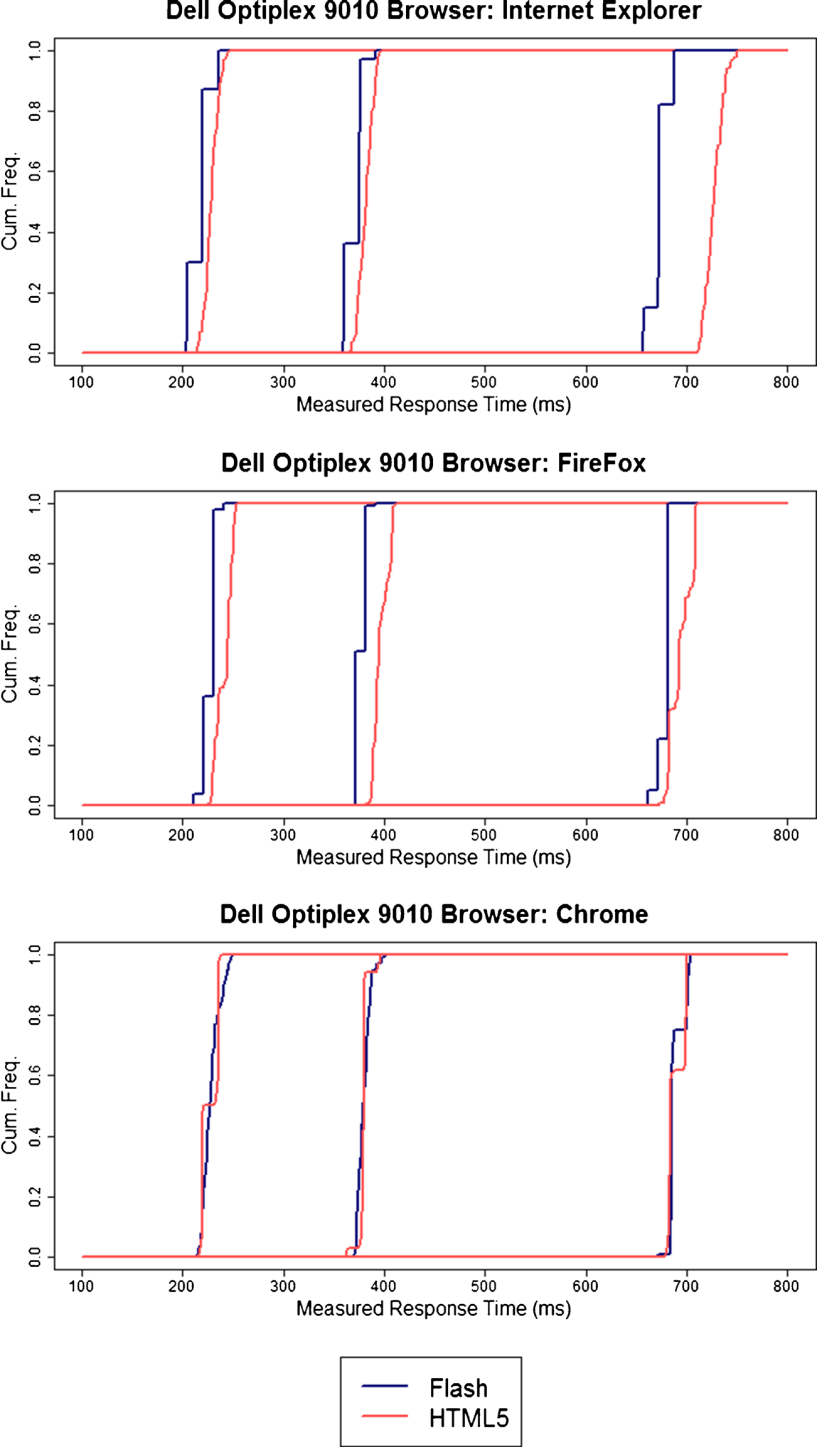
Cumulative frequencies of measured response times on a single machine, for actual response times of 150, 300, and 600 ms, as a function of Web browser and Flash/HTML5 implementation

It is also useful to examine the distribution of response times, to see whether it appears normally distributed, or whether, as Neath et al. (2011) and Reimers and Stewart (2007) found, quantizing is seen, with a large number of observations of a particular measured response time, or actual display time, but occurrences of neighboring values. We found clear evidence in many cases for quantizing in both Flash and HTML5. However, the circumstances under which this occurred seemed to vary by machine, and by browser on the same machine. Figure 1 shows the cumulative distributions of response times for one machine with various browsers (similar plots for all machines tested can be seen in the Supplementary Materials). For Internet Explorer, there is a clear step-like function for the Flash response times, suggesting substantial quantizing, which is absent from the HTML5 distribution. Conversely, on the same computer, running the same code under Chrome shows more quantizing for HTML5 than for Flash. There were some obvious idiosyncrasies. For example, we did note that across several tests using Firefox, response times measured by Flash were multiples of 10 ms, something that we did not observe for other browsers on the same system. Some quantizing may be attributable to the fact the Flash uses movie-like frames for its display updates, which are not synchronized with screen refreshes.

**Fig. 2:**
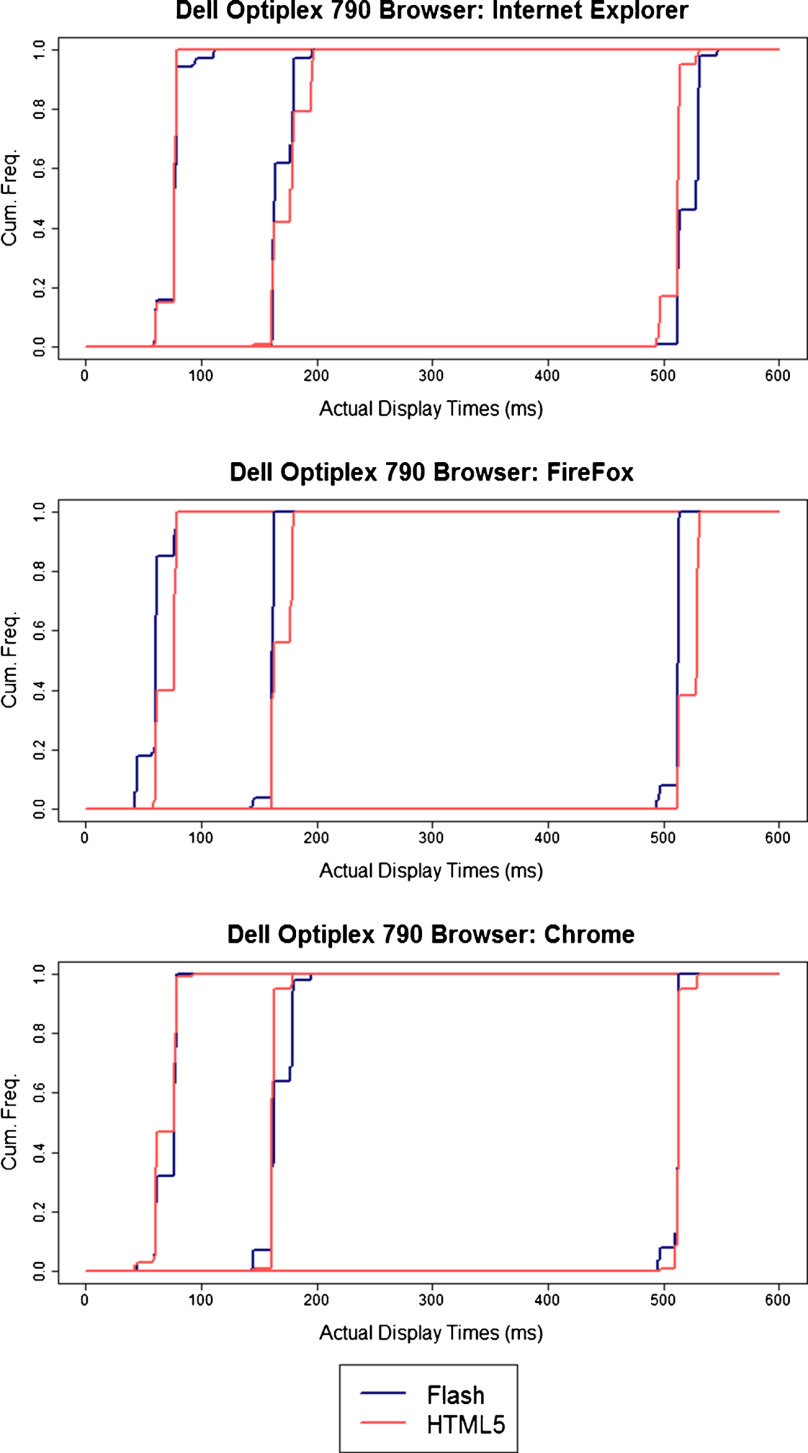
Cumulative frequencies of actual display durations on a single machine, for intended durations of 50, 150, and 500 ms, as a function of Web browser and Flash/HTML5 implementation

For stimulus display durations (Fig. 2), similar quantizing can be seen for both Flash and HTML5. Some quantizing would be expected, since the visual stimulus must remain on the screen for a fixed number of refreshes, so a monitor refreshing at a 100-Hz rate would show quantizing of display durations to the nearest 10 ms. Similarly, keyboard responses are polled intermittently. It is therefore possible that the quantizing observed here is not related to Flash or HTML5 per se.

## Discussion

The results of this systematic evaluation of different potential sources of variance in response times and stimulus durations across participants showed a number of things. First, all systems overestimated response times, by at least 30 ms. Second, all systems presented visual stimuli for longer than specified, sometimes by over 50 ms, although more generally by around 10–20 ms. Third, within-system variability appears to have been small, with standard deviations in presentation durations or recorded response times generally around 10 ms. Fourth, Flash and HTML5 gave, overall, fairly similar timing results. The only consistent difference that we noted was that display durations tended to be longer for HTML5 run on Internet Explorer, at least on less powerful systems. Fifth, apart from this, there were minimal differences between browsers in recorded response times and presentation durations. Sixth, we found nontrivial differences across systems in measured response times and duration timings.

Overall, these findings suggest that within-subjects Web-based experiments should not be constrained by technical limitations. All of the systems we tested showed low standard deviations for response times, meaning that minimal power would be lost by the random noise introduced by the system. In fact, the standard deviations measured here were often not much larger than those we obtained using the psychological testing software E-Prime.

The results also suggest that between-subjects experimental results will be largely unaffected by the choice of browser or the time duration to be measured. However, the results do suggest that there may be more substantial variability across systems. This means that between-subjects experiments may be subject to greater noise, and may have implications for correlational studies in which system performance is confounded with other variables of interest. In the present results we examined only four systems. To get a better idea of the cross-system variability of timing performance in Flash and HTML5, we examined timing on a further 15 systems.

## Study 2

In Study 1, we found differences in measured response times and, to a lesser degree, stimulus presentation durations, across the four systems tested, but it is unclear how representative this variability might be of the general Web-experiment-participating public. This second study expanded on the results of Study 1 by running a series of shorter timing tests on a larger number of PCs. By examining a greater number of PCs, which were selected to vary in Windows operating system version, processing speed, graphics capability, and memory, it would be possible to start to determine how much between-system variability we should expect in Web-based research, and the sources of some of this variability.

In this study, we repeated the measures used in Study 1: visual display duration and response time measurement. However, given the relatively small effect of duration, and the fact that we have documented it already, we used just a single duration for these wider tests, selecting the middle durations from Study 1: 150 ms for presentation, and 300 ms for response time. Since many of the machines tested in Study 2 were borrowed from friends and colleagues, we did not want to install new software onto the machine, and only used the browser installed on the machine. For each machine in this study, we ran each test of 100 trials three times and took the average of the means and *SD*s.

Details of the machines used, and their specs, can be seen in Table 6.

## Test results

Deviations from the 300-ms response time and 150-ms presentation duration are given in Table 6. All systems overestimated response times, and all systems displayed stimuli for longer than specified. The correlation between overestimation in Flash and overestimation in HTML5 was .82, *p* < .001, and the correlation between overpresentation in the two systems was .64, *p* = .003. There was no correlation between overestimation and overpresentation across the systems tested. These results suggest that the cross-system variability is mainly due to factors common to both Flash and HTML5, rather than specific to each of the program implementations.

**Fig. 3:**
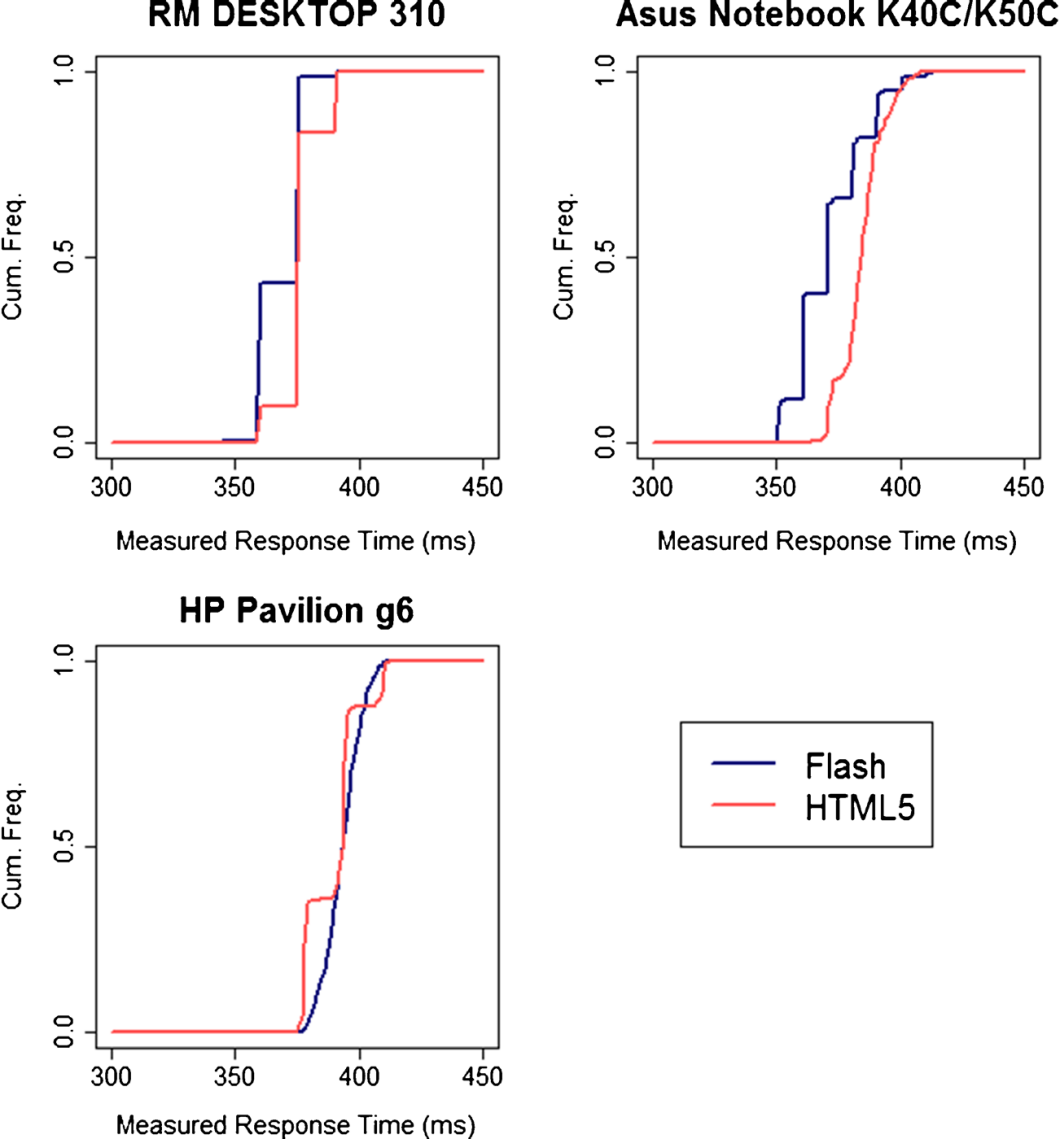
Cumulative frequencies of measured response times across three machines, for an actual 300-ms response time, as a function of Flash/HTML5 implementation

**Fig. 4:**
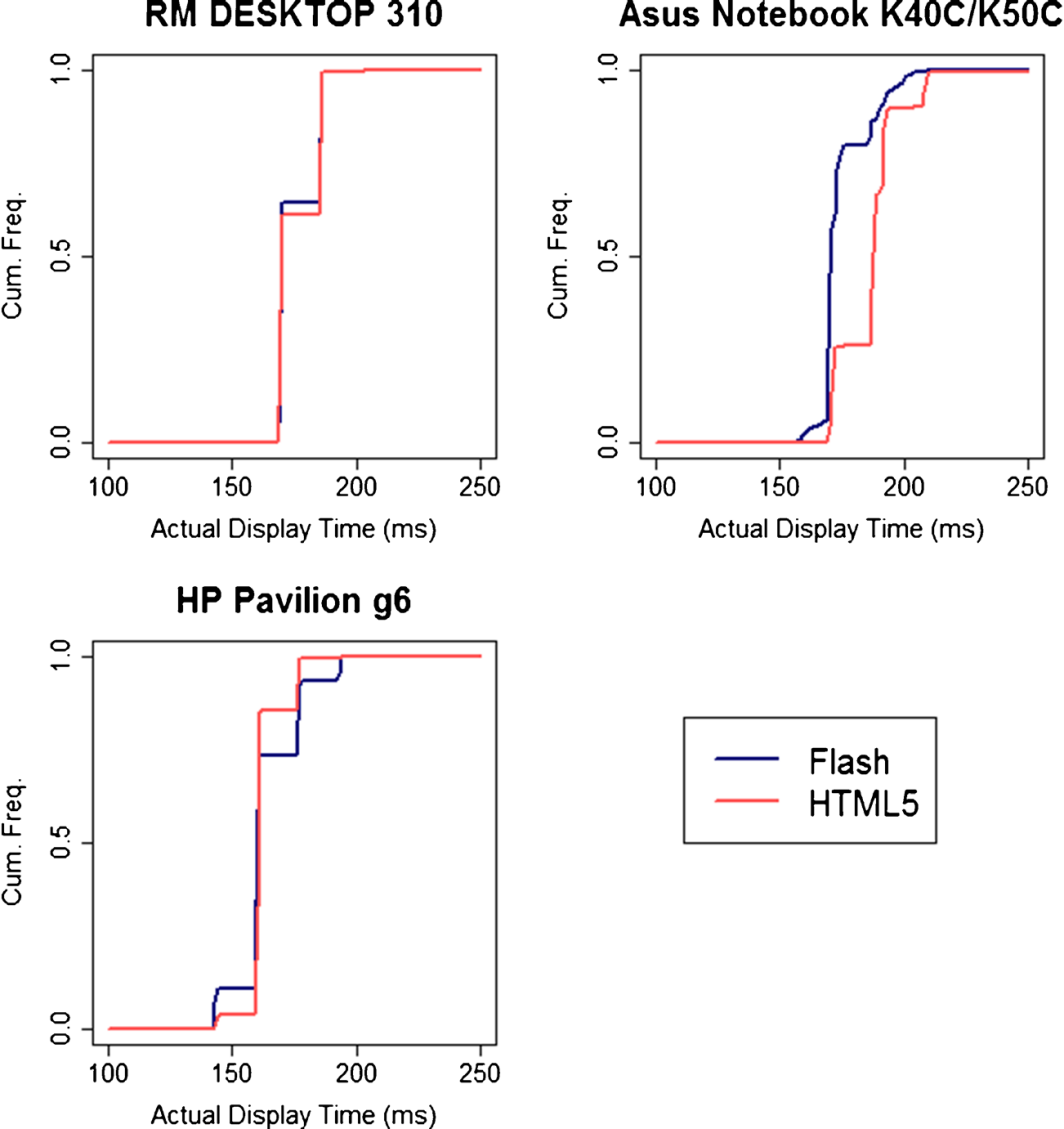
Cumulative frequencies of actual display durations across three machines, for an intended duration of 150 ms, as a function of Flash/HTML5 implementation

Quantizing varied significantly across systems. We give three examples of different patterns found for response time measurements (Fig. 3) and display durations (Fig. 4). Distributions for all systems can be seen in the Supplementary Materials. As before, we could detect no systematic predictors of smooth or quantized timings.

### Modeling

The measurement error added by Flash and HTML5 does not make much difference to the power of typical experiments. For a worst-case scenario, we assumed a two-condition between-subjects design because, in this case, the large between-computer differences in the systematic overestimation of each response time and the small within-computer trial-to-trial fluctuations would both obscure an effect of condition. (For a within-subject design, only the latter variability would matter.)

One hundred response times were drawn from exponentially modified Gaussian distributions (ex-Gaussians) to simulate 100 trials from each participant. Each simulated participant was assumed to have a different ex-Gaussian: The mean of the Gaussian component was drawn from a uniform distribution between 400 and 600 ms, the standard deviation of the Gaussian component was drawn from a uniform distribution between 25 and 75 ms, and the mean of the exponential component was drawn from a uniform distribution between 50 and 100 ms. The effect of condition was assumed to differ across participants and was drawn from a uniform distribution of between 0 and 100 ms, giving an overall population mean effect size of 50 ms. The assumption of an ex-Gaussian distribution for response times and these parameter values are typical in modeling response time distributions (e.g., Brand & Bradley, 2012).

**Fig. 5:**
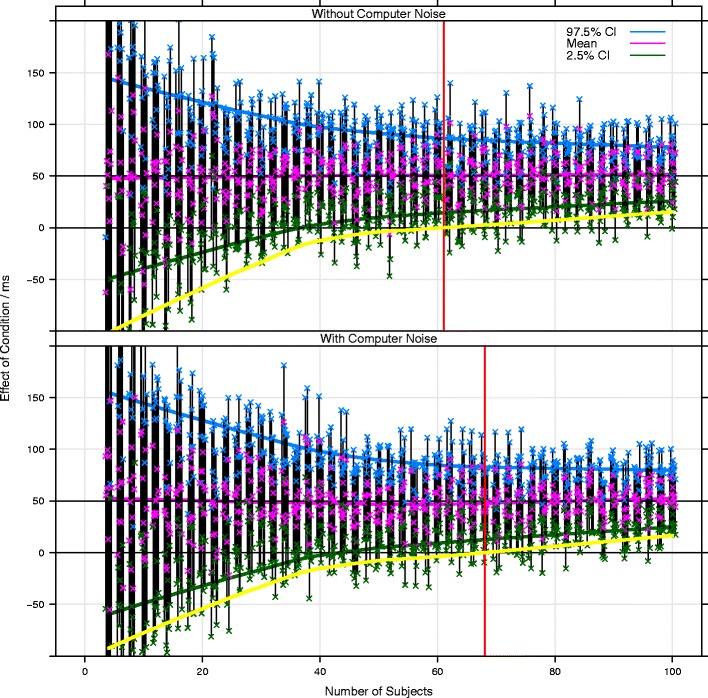
Effect of Flash/HTML5 measurement error on the detection of a 50-ms effect in a between-subjects design. Each vertical black bar represents a 95 % confidence interval for the estimated effect size from a simulated experiment. The pink crosses mark the estimated means, and the blue and green crosses mark the extents of the 95 % confidence interval for each mean. The simulations were repeated ten times at each sample size. The pink, blue, and green lines are loess-smoothed estimates of the means of the estimated means and confidence intervals. The yellow lines are the 20th percentiles for the lower ends of the confidence intervals, so by definition, 80 % of the confidence intervals are completely above this line. The red vertical lines mark the points at which 80 % of the confidence intervals are above zero (i.e., where the yellow line crosses zero) and indicate the sample sizes beyond which 80 % or more of experiments would give a significant effect (i.e., where 80 % or more of the confidence intervals do not contain zero). The upper-panel simulations assume millisecond-accurate measurement of response times. The lower-panel simulations assume Flash/HTML5 measurement error as we measured here

The top panel of Fig. 5 plots the results of many simulations of the experiment, assuming millisecond-accurate recording of response times. Each vertical black line is a 95 % confidence interval for the estimate of the effect of condition from a single simulated experiment. The pink crosses mark the estimated means, and the blue and green crosses mark the ends of the 95 % confidence intervals for that means. The simulation was repeated ten times at each sample size, with sample size (on the *x*-axis) ranging from four to 100 participants. The solid pink, green, and blue lines mark the loess-smoothed means of the means and confidence intervals, and converge at larger sample sizes. The yellow line is the loess-smoothed 20th percentile for the lower end of the confidence intervals. By definition, 80 % of confidence intervals fall completely above this line. Of central interest is the point at which this yellow line crosses zero, marked by a red vertical line at about 62 subjects. This is the sample size required for 80 % or more of the simulated experiments to give 95 % confidence intervals that do not include zero (i.e., are significant at the 5 % level in a two-tailed test).

The lower panel of Fig. 5 repeats the simulation, but assuming Flash/HTML5 measurement error. We assumed that each simulated participant’s simulated computer added a constant to all of their response times, which was drawn from a uniform distribution between 30 and 100 ms. We also assumed that each simulated participant’s simulated computer added random Gaussian noise to each trial, with a standard deviation drawn from a uniform distribution between 6 and 17 ms. These ranges match those we observed in our testing, shown in Table 6. This extra variability makes only a small difference to the number of participants required for 80 % power. Seven more participants were required (an increase of about 10 %), and had a more conventional within-subjects design been used, eliminating the effect of the systematic overestimation of response times, there would be no effective difference. The cost of using Flash/HTML5 to measure differences in response times is low (see Brand & Bradley, 2012, for a similar, theoretical argument).

## General discussion

Two studies used specialist hardware to examine variability in stimulus presentation durations and measured response times under Flash and a setup using HTML5, CSS3, and JavaScript, particularly differences between the two implementations, effects of stimulus duration, browser, and system under which the experiment was run. In general, within-system reliability was very good for both Flash and HTML5—standard deviations in measured response times and stimulus presentation durations were generally less than 10 ms. External validity was less impressive, with overestimations of response times of between 30 and 100 ms, depending on system. The effect of browser was generally small and nonsystematic, although presentation durations with HTML5 and Internet Explorer tended to be longer than in other conditions. Similarly, stimulus duration and actual response time were relatively unimportant—actual response times of 150-, 300-, and 600-ms response times gave similar overestimations. Central or graphics processing power appeared to have no systematic effect on timing performance: The most accurate system for response measurement and presentation accuracy was an old Windows XP machine. Although our sample was too small for strong conclusions to be drawn, it is interesting to note that the two systems running Windows XP gave much more accurate measurements of response time than virtually all the more powerful Windows Vista and 7 systems. This gives credence to Plant and Quinlan’s (2013) argument that more up-to-date, complex operating systems may actually lead to worse performance in measuring behavioral data.

### Implications for Web-based research

With regard to stimulus presentation durations, we would warn against running experiments in which precise stimulus durations are required, particularly those of short duration. Our findings suggest that durations tend to be longer than specified, and that this overpresentation can vary from ~5 to ~25 ms. We also note that on rare occasions, durations can be up to 100 ms longer than specified. Clearly, this would affect, for example, masked priming experiments, and may explain the results of Crump et al. (2013), who failed to replicate well-established findings for very short durations. As such, we would not recommend running experiments that require precise stimulus durations online using Flash or HTML5. On the other hand, for most experiments, variability in presentation durations of up to 25 ms would not be a problem, particularly with such good within-system reliability, meaning Web-based research in these situations should be fine.

For measuring response times, the implications of these findings depend on the design of experiment used. It is very rare simply to wish to measure in absolute terms how quickly someone responds to a stimulus—and in those cases in which such a measurement was required we would definitely discourage the use of Web-based research. The results here indicate that such a measurement would overestimate the response time by 30–100 ms. A between-subjects comparison of response times across two conditions controls for this overestimate and the extra noise from Flash/HTML5 can be offset by running about 10 % more participants. And a within-subjects comparison of response times across two conditions is almost unaffected.

Correlational studies are potentially more problematic. Experiments examining correlations between response times on two different tasks would risk finding spurious false positives from system-to-system variability. More generally, if system properties affecting response time measurement are correlated with demographics of interest, such as gender, age, education or income, or, say, personality measures (e.g., Buchanan & Reips, 2001, found differences in personality between PC and Mac users), then spurious relationships between demographics or personality and response times could be detected. Similarly, longitudinal research could be problematic online, both for finding general trends over time, which would be affected by software and hardware development, and comparing groups’ longitudinal changes in response times, if people of different demographics upgrade more or less frequently, or switch to different types of machine. It would also be the case that studies such as clinical trials, which are not longitudinal in design but may last several years, may be affected by upgrades to hardware or operating system during the trial. This may, of course, also be the case for lab-based research in which hardware or software are updated during a longitudinal study.

### Limitations of present research

The research reported here is a fairly comprehensive attempt to measure how response times and stimulus presentation durations vary across systems. However, it is not exhaustive. Our selection of systems was based on a convenience sample, and is not completely representative of the sample of computers on which Web-based experiments are completed. We were not able to deconfound operating system and hardware, as more recent systems tended to have more powerful hardware and more recent versions of Windows running. Nor were we able to test all possible configurations—it is almost inevitable that some untested combinations of hardware, software, and running applications would produce much less reliable results than those reported here. We did not account for variability in input devices (see, e.g., Plant, Hammond, & Whitehouse, 2003), since we were able only to dismantle a single USB keyboard for the response time tests. There is, of course, likely to be variability in timing measured across keyboards (and between, e.g., keyboards, touchscreens, and mice).

A second issue is that it is not clear whether the variability and patterns of quantizing seen across systems are stable phenomena based on hardware or configuration differences, or transient differences that occurred because the two systems were performing different background tasks at the points at which we ran the tests. To discover the cause of the cross-system variability, this would need to be determined. However, since our aim was to estimate variability, and hence the feasibility of running response-time-based Web experiments, this was less important here.

Finally, we note that these studies used simple stimuli, coded as cleanly as possible. It is possible that the use of different methods to record timings or to control stimulus presentation could lead to a different pattern of results. Similarly, the use of large graphics files for visual stimuli could lead to worse timing performance, or to more of an effect of system capabilities on timing performance. More generally, we suggest that researchers should be mindful of the fact that their results are likely to be distortions of the actual response times.

While taking these limitations into account, we have reason to be cautiously positive about measuring response times through a Web browser using Flash or HTML5. Of course, this is a conclusion that could change rapidly, as new operating systems are released, along with new browsers, new versions of Flash, and new input and display devices. As such, this study can only provide a snapshot.

Of course, other sources of participant-generated variance are likely to be larger for Web-based studies than for lab-based studies—for example, lack of attentiveness, misunderstanding of instructions, distractions, motivational limitations, and so on. These are more general issues that researchers must consider when designing and implementing Web-based experiments. We also note that these findings apply specifically to Web-based experiments that use simple visual stimuli. However, it seems from this research that technical variability across systems and within-system noise are likely to make relatively minor contributions to overall variance.

## Electronic supplementary material

Below is the link to the electronic supplementary material.

ESM 1 (ZIP 1.07 MB)

## References

[CR1] Birnbaum MH, Birnbaum M (2000). Decision making in the lab and on the Web. Psychological experiments on the Internet.

[CR2] Brand A, Bradley MT (2012). Assessing the effects of technical variance on the statistical outcomes of web experiments measuring response times. Social Science Computer Review.

[CR3] Buchanan, T., & Reips, U.-D. (2001). Platform-dependent biases in online research: Do Mac users really think different? In K. J. Jonas, P. Breuer, B. Schauenburg, & M. Boos (Eds.), *Perspectives on Internet research: Concepts and methods* (pp. 1–11). Retrieved from http://www.uni-konstanz.de/iscience/reips/pubs/papers/Buchanan_Reips2001.pdf

[CR4] Chandler, J., Mueller, P., & Paolacci, G. (2013). Nonnaïveté among Amazon Mechanical Turk workers: Consequences and solutions for behavioral researchers. *Behavior Research Methods, 46,* 112–130 10.3758/s13428-013-0365-7 23835650

[CR5] Crump, M. J., McDonnell, J. V., & Gureckis, T. M. (2013). Evaluating Amazon’s Mechanical Turk as a tool for experimental behavioral research. *PLoS ONE, 8,* e57410 10.1371/journal.pone.0057410 PMC3596391 23516406

[CR6] Damian, M. F. (2010). Does variability in human performance outweigh imprecision in response devices such as computer keyboards? *Behavior Research Methods, 42,* 205–211 10.3758/BRM.42.1.205 20160300

[CR7] Kassam KS, Morewedge CK, Gilbert DT, Wilson TD (2011). Winners love winning and losers love money. Psychological Science.

[CR8] Keller, F., Gunasekharan, S., Mayo, N., & Corley, M. (2009). Timing accuracy of Web experiments: A case study using the WebExp software package. *Behavior Research Methods, 41,* 1–12 10.3758/BRM.41.1.12 19182118

[CR9] Kinderman, P., Schwannauer, M., Pontin, E., & Tai, S. (2013). Psychological processes mediate the impact of familial risk, social circumstances and life events on mental health. *PLoS ONE, 8,* e76564. doi:10.1371/journal.pone.0076564 10.1371/journal.pone.0076564 PMC3797803 24146890

[CR10] Mason, W., & Suri, S. (2012). Conducting behavioral research on Amazon’s Mechanical Turk. *Behavior Research Methods, 44,* 1–23 10.3758/s13428-011-0124-6 21717266

[CR11] Musch J, Reips UD, Birnbaum M (2000). A brief history of Web experimenting. Psychological experiments on the Internet.

[CR12] Neath I, Earle A, Hallett D, Surprenant AM (2011). Response time accuracy in Apple Macintosh computers. Behavior Research Methods.

[CR13] Plant, R. R., Hammond, N., & Turner, G. (2004). Self-validating presentation and response timing in cognitive paradigms: How and why? *Behavior Research Methods, Instruments, & Computers, 36,* 291–303 10.3758/bf03195575 15354695

[CR14] Plant, R. P., Hammond, N., & Whitehouse, T. (2003). How choice of mouse may affect response timing in psychological studies. *Behavior Research Methods, Instruments, & Computers, 35,* 276–284 10.3758/bf03202553 12834085

[CR15] Plant, R. R., & Quinlan, P. T. (2013). Could millisecond timing errors in commonly used equipment be a cause of replication failure in some neuroscience studies? *Cognitive, Affective, & Behavioral Neuroscience, 13,* 598–614 10.3758/s13415-013-0166-6 23640111

[CR16] Reimers S (2007). The BBC Internet study: General methodology. Archives of Sexual Behavior.

[CR17] Reimers S (2009). A paycheck half-empty or half-full? Framing, fairness and progressive taxation. Judgment and Decision Making.

[CR18] Reimers, S., & Maylor, E. A. (2005). Task switching across the life span: Effects of age on general and specific switch costs. *Developmental Psychology, 41,* 661–671 10.1037/0012-1649.41.4.661 16060812

[CR19] Reimers S, Stewart N (2007). Adobe Flash as a medium for online experimentation: A test of reaction time measurement capabilities. Behavior Research Methods.

[CR20] Reimers S, Stewart N (2008). Using Adobe Flash Lite on mobile phones for psychological research: Reaction time measurement reliability and interdevice variability. Behavior Research Methods.

[CR21] Reips, U.-D., & Neuhaus, C. (2002). WEXTOR: A Web-based tool for generating and visualizing experimental designs and procedures. *Behavior Research Methods, Instruments, & Computers, 34,* 234–240. 10.3758/bf03195449 12109018

[CR22] Rosenberg, J. (2012). Google Play hits 25 billion downloads. Retrieved September 26, 2013, from http://officialandroid.blogspot.ca/2012/09/google-play-hits-25-billion-downloads.html.

[CR23] Schubert, T. W., Murteira, C., Collins, E. C., & Lopes, D. (2013). ScriptingRT: A software library for collecting response latencies in online studies of cognition. *PLoS ONE, 8,* e67769. doi:10.1371/journal.pone.0067769 10.1371/journal.pone.0067769 PMC3689727 23805326

[CR24] Simcox, T., & Fiez, J. A. (2014). Collecting response times using Amazon Mechanical Turk and Adobe Flash. *Behavior Research Methods, 46,* 95–111 10.3758/s13428-013-0345-y PMC5283577 23670340

[CR25] Stewart, N., Reimers, S., & Harris, A. J. L. (2014). On the origin of utility, weighting, and discounting functions: How they get their shapes and how to change their shapes. *Management Science*. doi:10.1287/mnsc.2013.1853

[CR26] Winokur, D. (2011). Flash to focus on PC browsing and mobile apps; Adobe to more aggressively contribute to HTML5. Retrieved, September 26, 2013 from http://blogs.adobe.com/conversations/2011/11/flash-focus.html

