# Supplementary material for: Presentation and response timing accuracy in Adobe Flash and HTML5/JavaScript Web experiments
Source: Behav Res Methods. 2014 Jun 6;47(2):309–27. doi: 10.3758/s13428-014-0471-1 (PMC4427652; doi:10.3758/s13428-014-0471-1)
Supplement: Supplementary file 1 — (ZIP 1.07 MB) [file 13428_2014_471_MOESM1_ESM.zip › Supplementary/Code/HTML5_code/square_RT/index.html]

# Experiment

Visual presentation accuracy.

View full screen

Start

Click me  
(or press any key)

# Thank you!

The test is over. Here are the reaction times:

BLANK
