# Supplementary material for: Presentation and response timing accuracy in Adobe Flash and HTML5/JavaScript Web experiments
Source: Behav Res Methods. 2014 Jun 6;47(2):309–27. doi: 10.3758/s13428-014-0471-1 (PMC4427652; doi:10.3758/s13428-014-0471-1)
Supplement: Supplementary file 1 — (ZIP 1.07 MB) [file 13428_2014_471_MOESM1_ESM.zip › Supplementary/Graphs/supp_study1_disp.pdf]

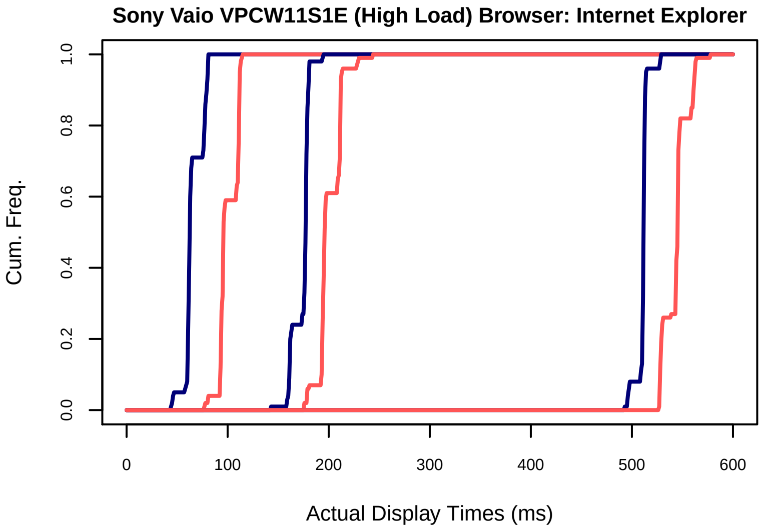
Sony Vaio VPCW11S1E (High Load) Browser: Internet Explorer
Cum. Freq.
0.0 0.2 0.4 0.6 0.8 1.0
0 100 200 300 400 500 600
Actual Display Times (ms)

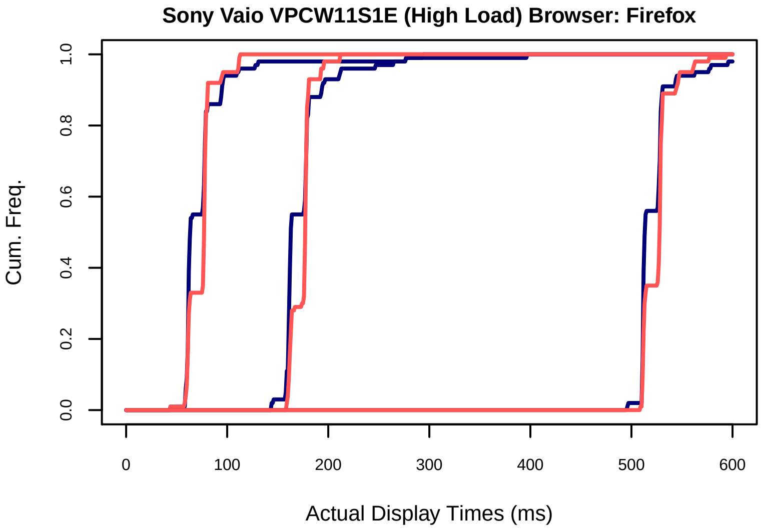
Sony Vaio VPCW11S1E (High Load) Browser: Firefox
Cum. Freq.
0.0 0.2 0.4 0.6 0.8 1.0
0 100 200 300 400 500 600
Actual Display Times (ms)

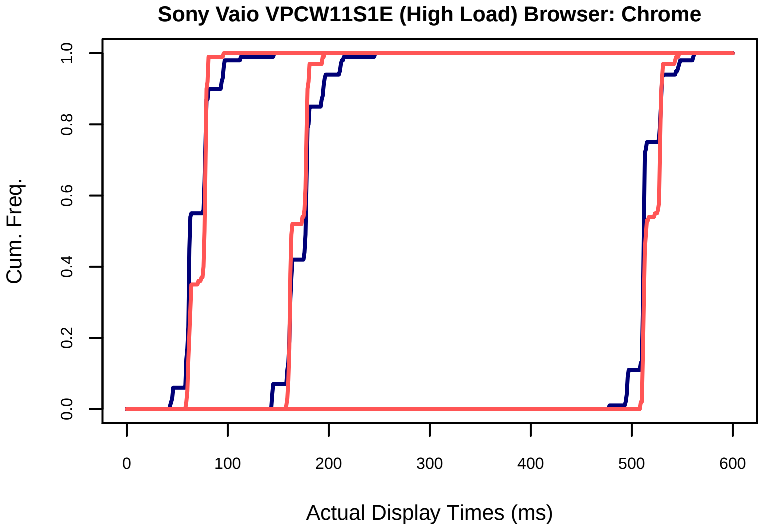
Sony Vaio VPCW11S1E (High Load) Browser: Chrome
Cum. Freq.
0.0 0.2 0.4 0.6 0.8 1.0
0 100 200 300 400 500 600
Actual Display Times (ms)

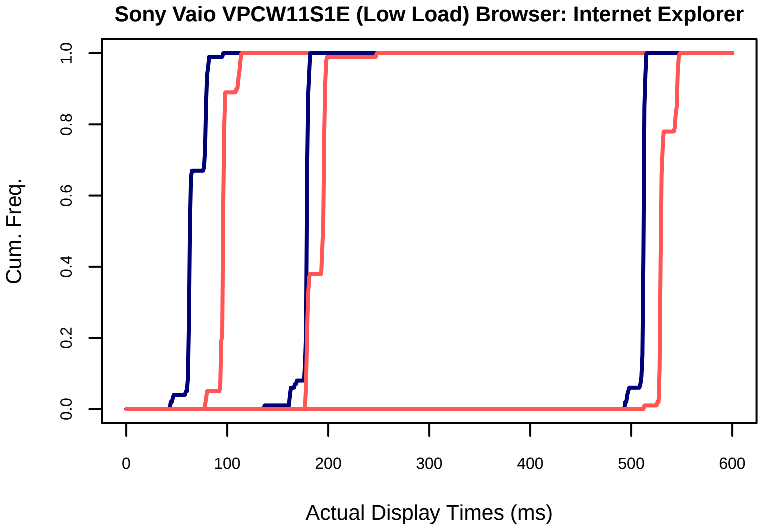
Sony Vaio VPCW11S1E (Low Load) Browser: Internet Explorer
Cum. Freq.
0.0 0.2 0.4 0.6 0.8 1.0
0 100 200 300 400 500 600
Actual Display Times (ms)

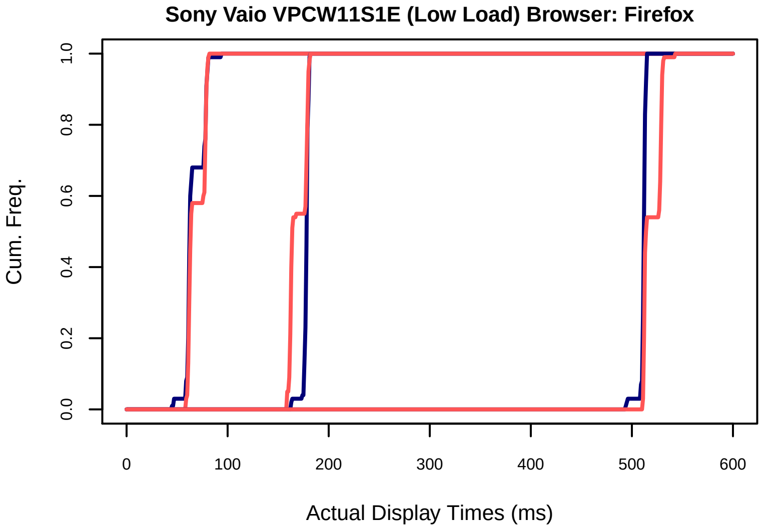
Sony Vaio VPCW11S1E (Low Load) Browser: Firefox
Cum. Freq.
0.0 0.2 0.4 0.6 0.8 1.0
0 100 200 300 400 500 600
Actual Display Times (ms)

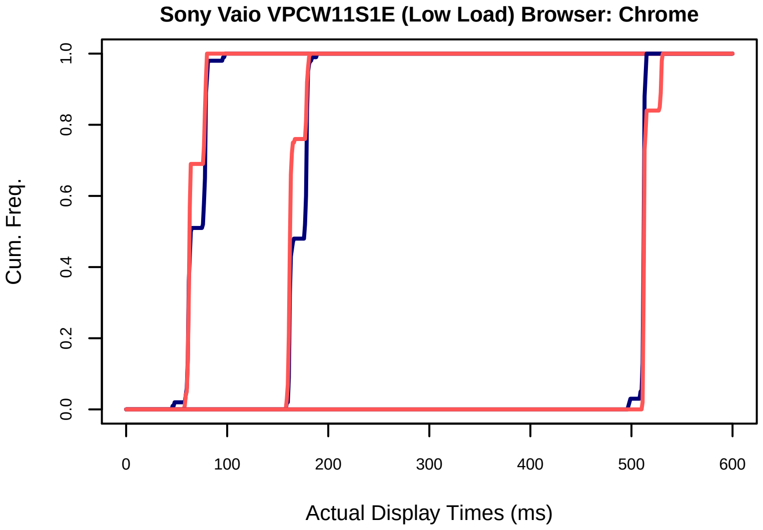
Sony Vaio VPCW11S1E (Low Load) Browser: Chrome
Cum. Freq.
0.0 0.2 0.4 0.6 0.8 1.0
0 100 200 300 400 500 600
Actual Display Times (ms)

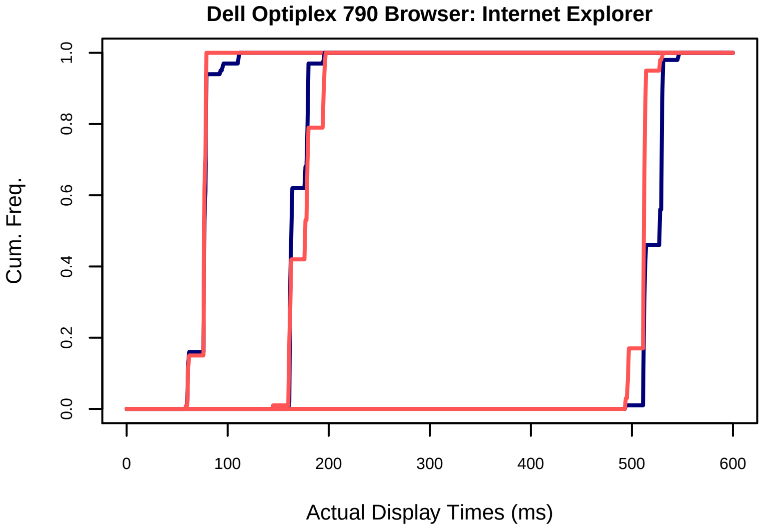
Dell Optiplex 790 Browser: Internet Explorer
Cum. Freq.
0.0 0.2 0.4 0.6 0.8 1.0
0 100 200 300 400 500 600
Actual Display Times (ms)

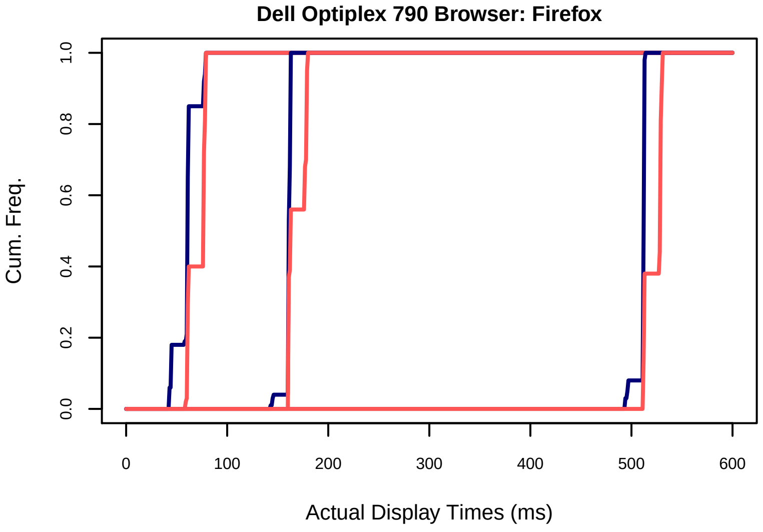
Dell Optiplex 790 Browser: Firefox
Cum. Freq.
0.0 0.2 0.4 0.6 0.8 1.0
0 100 200 300 400 500 600
Actual Display Times (ms)

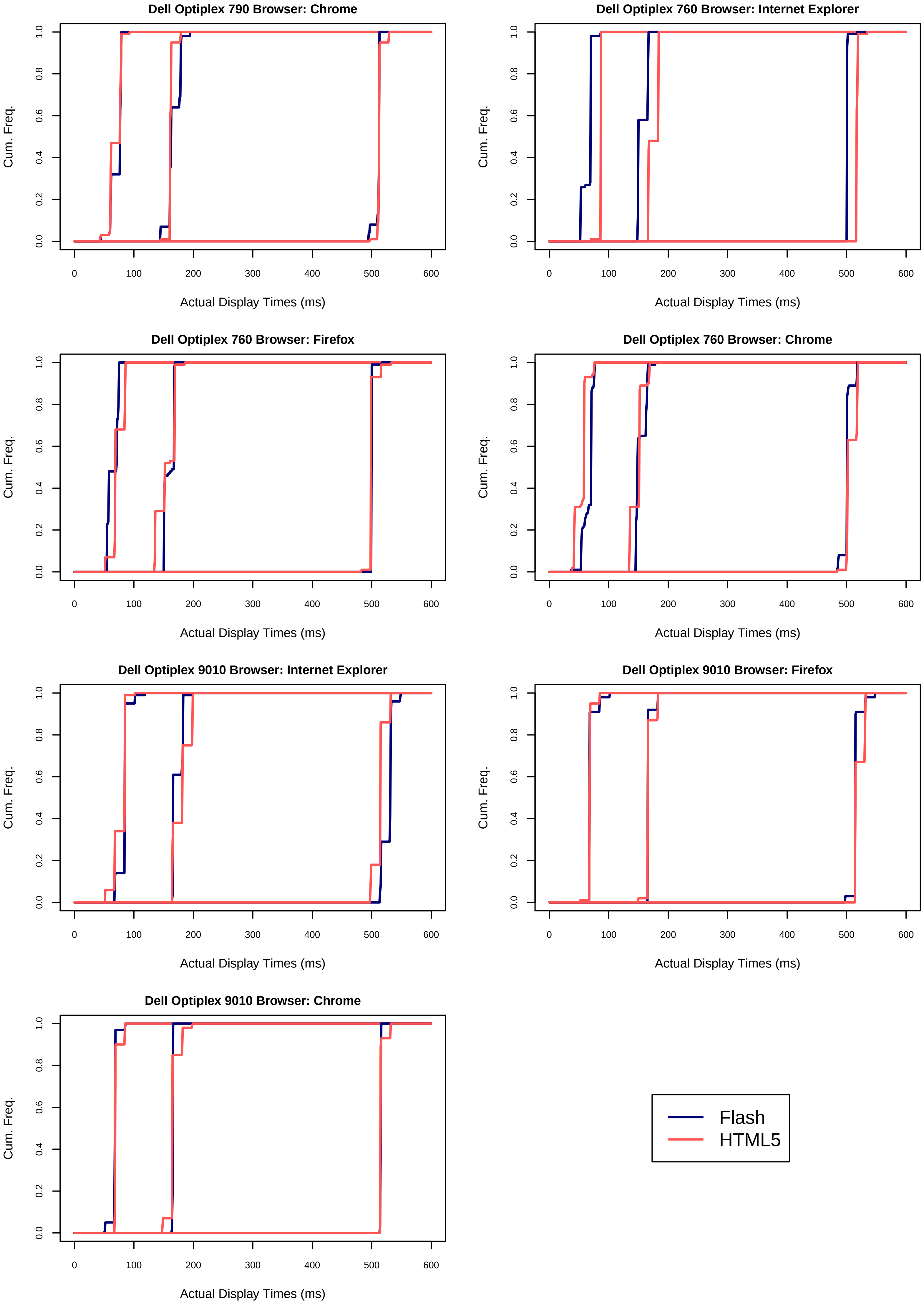

Dell Optiplex 790 Browser: Chrome
Dell Optiplex 760 Browser: Internet Explorer
Dell Optiplex 760 Browser: Firefox
Dell Optiplex 760 Browser: Chrome
Dell Optiplex 9010 Browser: Internet Explorer
Dell Optiplex 9010 Browser: Firefox
Dell Optiplex 9010 Browser: Chrome
Cum. Freq.
0.0
0.2
0.4
0.6
0.8
1.0
Actual Display Times (ms)
0
100
200
300
400
500
600
Flash
HTML5
