# Supplementary material for: Presentation and response timing accuracy in Adobe Flash and HTML5/JavaScript Web experiments
Source: Behav Res Methods. 2014 Jun 6;47(2):309–27. doi: 10.3758/s13428-014-0471-1 (PMC4427652; doi:10.3758/s13428-014-0471-1)
Supplement: Supplementary file 1 — (ZIP 1.07 MB) [file 13428_2014_471_MOESM1_ESM.zip › Supplementary/Graphs/supp_study2_disp.pdf]

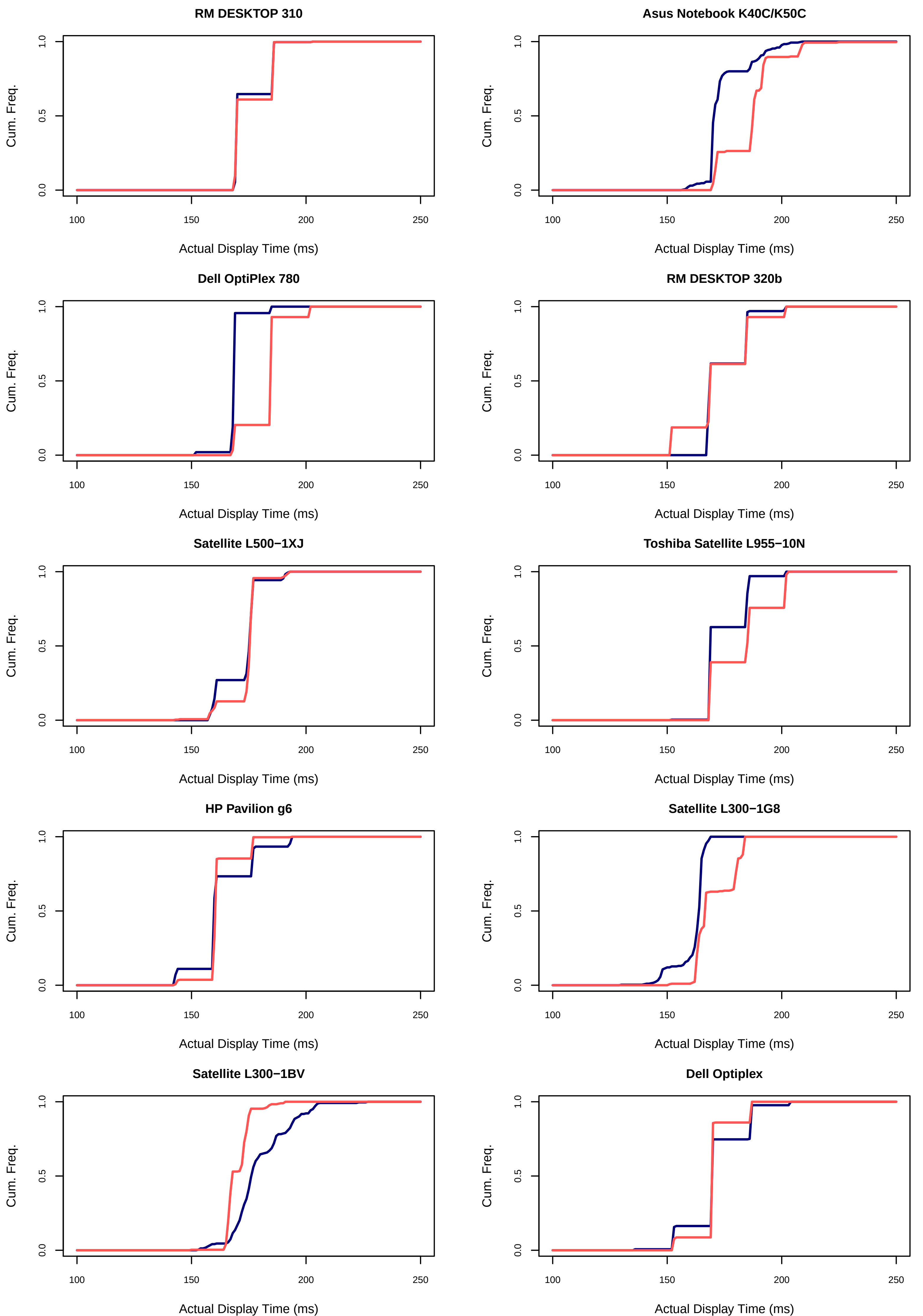
RM DESKTOP 310
Asus Notebook K40C/K50C
Dell OptiPlex 780
RM DESKTOP 320b
Satellite L500−1XJ
Toshiba Satellite L955−10N
HP Pavilion g6
Satellite L300−1G8
Satellite L300−1BV
Dell Optiplex
Cum. Freq.
0.0
0.5
1.0
100
150
200
250
Actual Display Time (ms)

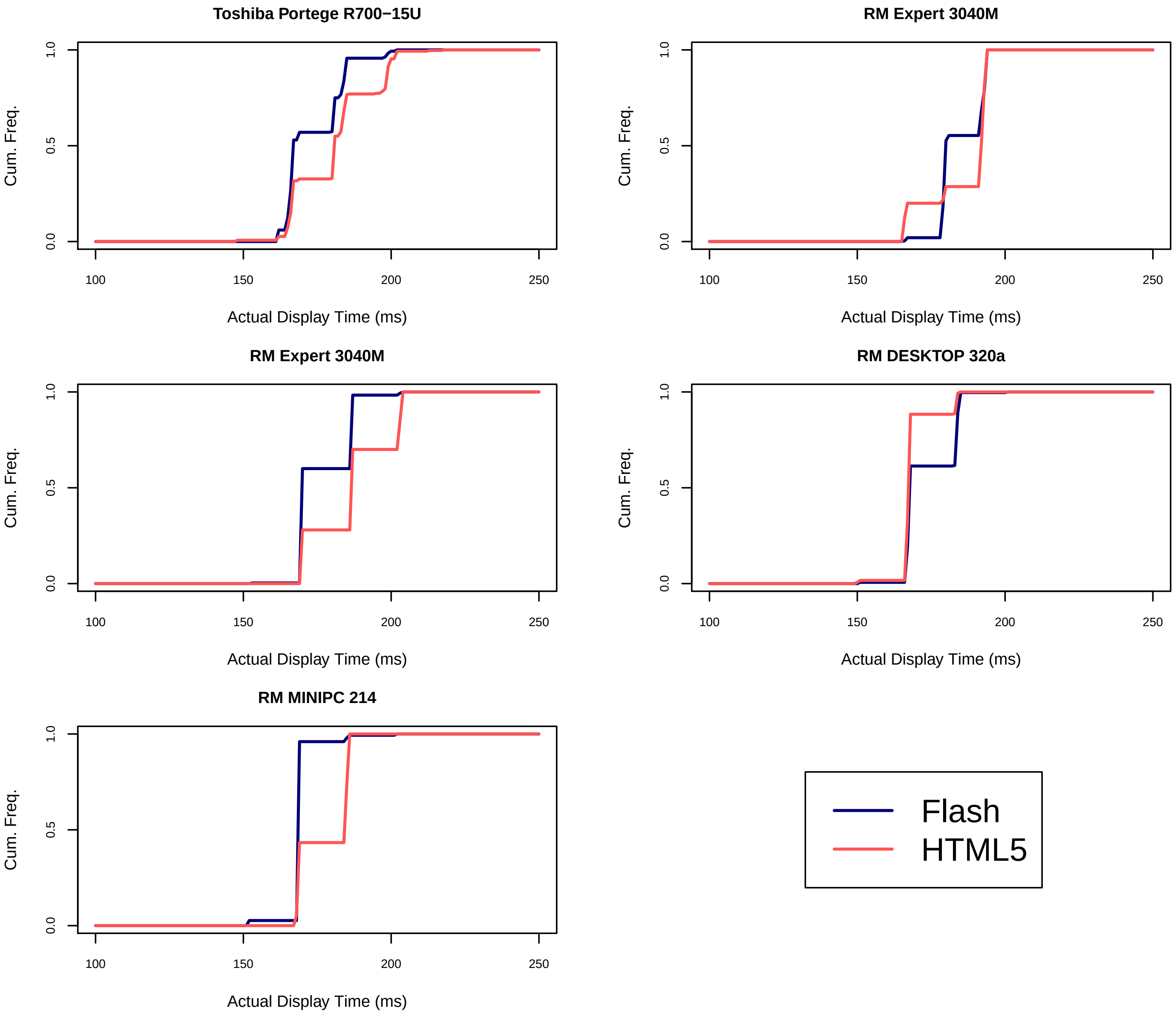
Toshiba Portege R700−15U
RM Expert 3040M
RM Expert 3040M
RM DESKTOP 320a
RM MINIPC 214
Cum. Freq.
0.0
0.5
1.0
100
150
200
250
Actual Display Time (ms)
Flash
HTML5
